# PHYRN: A Robust Method for Phylogenetic Analysis of Highly Divergent Sequences

**DOI:** 10.1371/journal.pone.0034261

**Published:** 2012-04-13

**Authors:** Gaurav Bhardwaj, Kyung Dae Ko, Yoojin Hong, Zhenhai Zhang, Ngai Lam Ho, Sree V. Chintapalli, Lindsay A. Kline, Matthew Gotlin, David Nicholas Hartranft, Morgen E. Patterson, Foram Dave, Evan J. Smith, Edward C. Holmes, Randen L. Patterson, Damian B. van Rossum

**Affiliations:** 1 Center for Computational Proteomics, The Pennsylvania State University, University Park, Pennsylvania, United States of America; 2 Department of Biology, The Pennsylvania State University, University Park, Pennsylvania, United States of America; 3 Department of Computer Science and Engineering, The Pennsylvania State University, University Park, Pennsylvania, United States of America; 4 Department of Biochemistry and Molecular Biology, The Pennsylvania State University, University Park, Pennsylvania, United States of America; 5 Fogarty International Center, National Institutes of Health, Bethesda, Maryland, United States of America; 6 Department of Biochemistry and Molecular Medicine, School of Medicine, University of California Davis, Davis, California, United States of America; 7 Department of Physiology and Membrane Biology, School of Medicine, University of California Davis, Davis, California, United States of America; 8 Center for Translational Bioscience and Computing, University of California Davis, Davis, California, United States of America; Tel Aviv University, Israel

## Abstract

Both multiple sequence alignment and phylogenetic analysis are problematic in the “twilight zone” of sequence similarity (≤25% amino acid identity). Herein we explore the accuracy of phylogenetic inference at extreme sequence divergence using a variety of simulated data sets. We evaluate four leading multiple sequence alignment (MSA) methods (MAFFT, T-COFFEE, CLUSTAL, and MUSCLE) and six commonly used programs of tree estimation (Distance-based: Neighbor-Joining; Character-based: PhyML, RAxML, GARLI, Maximum Parsimony, and Bayesian) against a novel MSA-independent method (PHYRN) described here. Strikingly, at “midnight zone” genetic distances (∼7% pairwise identity and 4.0 gaps per position), PHYRN returns high-resolution phylogenies that outperform traditional approaches. We reason this is due to PHRYN's capability to amplify informative positions, even at the most extreme levels of sequence divergence. We also assess the applicability of the PHYRN algorithm for inferring deep evolutionary relationships in the divergent DANGER protein superfamily, for which PHYRN infers a more robust tree compared to MSA-based approaches. Taken together, these results demonstrate that PHYRN represents a powerful mechanism for mapping uncharted frontiers in highly divergent protein sequence data sets.

## Introduction

Inferring phylogenetic history among highly divergent protein sequences is one of the most challenging problems in modern evolutionary biology. The ability to reliably determine the evolutionary history of protein sequences that fall below ∼25% identity (i.e. the “twilight zone” and lower still, the “midnight zone”) would allow for better identification of homologous proteins and shed light on key events in the deep evolutionary past [1], [2], [3]. To date, most attempts to resolve deep-node evolutionary relationships have relied upon improving methods, models, and parameters of multiple sequence alignment (MSA) and/or tree inference programs. However, MSA methods tend to get progressively worse with additional sequence divergence [4], [5]. This is due to the low information content of divergent sequences and the subsequent loss of informative points (i.e. number of common sites). No matter how robust a given tree-building algorithm performs, this lack of informative points tends to result in poor phylogenetic inference (i.e. noise in, noise out).

Current phylogenetic analysis often follows a two-step process: (i) obtain a guide tree based on percentage identity of all-against-all pairwise alignments, which designates the order of a progressive alignment, and (ii) estimate a phylogeny based upon the resultant MSA with distance-based or character-based tree inference programs. Distance methods (e.g. UPGMA, Neighbor Joining) are fast, and can handle large numbers of sequences [6]. However, distance-based trees are often erroneous when rates of substitution vary greatly among lineages. Thus, distance-based methods are generally thought to be inferior to character-based methods (e.g. Parsimony, Maximum Likelihood (ML), and Bayesian). Character-based inference can generate trees with the minimum number of changes needed to explain the data, or the highest likelihood of occurring with the given data and assuming a particular model of molecular evolution. The downside to character-based phylogenetic inference is the computational cost and problems with scalability to large data sets. Further, both distance- and character-based methods are prone to long branch attraction in which rapidly evolving sequences (with long branches), are placed with other rapidly evolving sequences even if they are not sister taxa [7].

We developed an alternative approach to MSA-based tree inference which utilizes the Euclidean distance of sequence profiles (a.k.a. phylogenetic profiles or NxM matrices) [8]. In this manner, the sequence profile of a set N of query amino acid sequences is defined as vectors where each entry quantifies the pairwise alignment between N queries and a set M of position specific scoring matrices (PSSMs) [3], [9], [10], [11]. Given this matrix, we calculate the Euclidean distance between all pairs and generate a NXN distance matrix for tree inference. The statistical robustness and computational cost of this initial algorithm did not make it feasible in practice; however, it was sufficiently robust in a benchmark data set of divergent retroelements [8]. This initial success led us to pursue this approach further, and alterations to the initial algorithm are discussed in detail in following sections. The underlying theory is that through the use of PSSMs, sequence profiling methods can amplify the signal (i.e. informative positions) contained in each sequence, handle large data sets, and give more refined distance measures.

In this study we address the performance of various traditional phylogenetic approaches and our new method presented here, PHYRN, in simulated data sets at extreme divergence levels. We use simulated data sets as the test bed of performance because unlike biological data sets, the true history of simulated sequence data is known and predefined. Knowledge of the true evolutionary history of the sequences under consideration makes it possible to quantify the performance of phylogenetic inference algorithms [12], [13], [14], [15], [16], [17]. Simulated data sets also allow us to evaluate our performance at multiple different divergence levels using many replicates. In this study, we have compared PHYRN to four leading MSA methods (MAFFT, T-COFFEE, ClustalW, and MUSCLE), two alignment-free methods (Average Common Substring(ACS) approach and Lempel-Ziv(LZ) Distance), and seven established methods for tree estimation (Distance-based: Neighbor-Joining, FastME; Character-based: PhyML, RAxML, Garli (all three of which utilize a maximum likelihood approach), Maximum Parsimony, and Bayesian).

While simulated data sets represent a powerful way to benchmark accuracy of a given algorithm, they may not incorporate all the underlying mechanisms of natural molecular evolution (e.g. translocations, rearrangements, recombination and/or inversions) [18], [19]. Therefore, it is informative to test PHYRN in a biologically relevant test bed. Accordingly, we also evaluate whether PHYRN is capable of providing informative measurements that could describe the evolutionary history of the highly divergent developmental DANGER superfamily. Based on the results from synthetic data sets data sets, and DANGER superfamily, we propose that: (i) high-resolution phylogenies can be built for protein families using PHYRN, (ii) these measurements have robust statistical support and inform intra- and inter-group relationships, and (iii) these measures can outperform traditional MSA-dependent tree inference methods.

## Methods

### Generation and Sequence Evolution of Synthetic Data Sets

We artificially generated protein sequences using SeqGen and ROSE simulation packages to test the performance of phylogenetic methods in highly divergent sequences [18], [19]. In both simulations, sequences are created from a common ancestor to produce a data set of known size, divergence, and history using a variety of tree shapes (e.g. biased vs. unbiased). In this *in silico* evolutionary process, an accurate phylogenetic history is recorded since the MSA is created simultaneously, thereby allowing us to test the reliability of different methods of tree inference at different levels of sequence divergence. Both simulation methods use PAM matrices [20], where increasing PAM score equates to decreasing percentage identity and similarity, and an increasing number of gaps. A key difference between SeqGen and ROSE is that SeqGen does not incorporate insertion-deletion events (indels) while generating these simulated protein families. ROSE does include indels, providing a better approximation of molecular evolution (see Supporting Methods S1 for more details).

Simulated sequences were then aligned by PHYRN or a distance estimation technique (MSA or alignment-free) and passed to the tree estimation method. The estimated trees are scored against the true tree for accuracy via two methods. First, we used the CONSENSE program in the PHYLIP v3.67 package (http://evolution.genetics.washington.edu/phylip.html) to generate consensus trees between the true-history tree and the estimated trees. Recapitulation rate and percentages were then calculated from consensus tree newick files. Deep nodes were defined as those which are evolutionary ancestors of last two tiers of leaf nodes. For a second measure of topological difference we used the ‘treedist’ program in the PHYLIP v3.67 package to calculate symmetric distances of Robinson and Foulds (RF distance) [21]. RF distance is a well-established metric for comparing tree topologies in which bipartitions between two trees are compared to calculate difference in their topologies. For two trees with exactly the same topology, this distance is 0, but for two trees of n leaves, with all branches differently placed, symmetric distance is equal to 2(n–3). Thus, the accuracy of a tree-building algorithm decreases with the symmetric distance score from the true simulated tree.

To simulate sequence evolution, a single amino acid sequence was placed at the root of the tree *T* and evolved down the tree according to the parameters of the simulation programs. In this way each leaf of *T* has a sequence. For the majority of experiments we generated simulated data sets comprised of 100 sequences with an average length of 450 amino acids. We used Seq-Gen v 1.3.2 [19] with PAM as the default substitution matrix and varied scaling factor from 0.1 to 1 to generate multiple replicates (n = 25) of the synthetic data sets with sequences at different divergence ranges. The SeqGen scaling factor scales the branch lengths of the input tree to a specified value before generating data set from the input tree. This changes the expected number of amino acid substitutions per site for each branch, and thus changes the overall divergence of the simulated tree. We also used ROSE v1.3 [18] with default settings to generate multiple replicates of true trees (n = 25) across a range of divergence. The extent of sequence divergence was varied across multiple replicates by changing the average ROSE distance parameter from 100 PAM to 700 PAM. Importantly, both ROSE and Seq-Gen employed a fixed substitution rate across all branches, such that we assume a strict molecular clock. All simulated data sets are available upon request or downloadable from www.ccp.psu.edu/downloads.

**Table 1:** Performance Comparison of Phylogenetic Inference Methods.

Alignment/ Alignment-free Method	Tree Inference Method	Settings and Parameters	ROSE Data Sets#			
			100	550	650	750
LZ	Neighbor-Joining		14(+/−48.16)	116.88(+/−18.03)	126.96(+/−6.98)	143.36(+/−7.34)
Average Common Substring(ACS)	Neighbor-Joining		7.36(+/−35.14)	106.96(+/−20.98)	116.8(+/−18.80)	122(+/−13.49)
MUSCLE	Maximum Parsimony (ProtPars)		0.15(+/−0.54)	49.61(+/−15.64)	91.69(+/−13.24)	106.15(+/−11.76)
MUSCLE	Maximum Parsimony (PAUP)		0(+/−0)	33.2(+/−12.19)	77.68(+/−9.27)	99.2(+/−11.05)
MUSCLE	Neighbor-Joining		0(+/−0)	41.44(+/−13.50)	82.24(+/−14.08)	94.96(+/−16.04)
MUSCLE	Neighbor-Joining	JTT, Gamma = 0.5	0(+/−0)	13.04(+/−10.31)	68.4(+/−23.08)	99.36(+/−22.63)
MUSCLE	FastME	JTT, Gamm = 0.5	0(+/−0)	12.64(+/−12.72)	66(+/−21.73)	92.96(+/−21.31)
MUSCLE	Neighbor-Joining	JTT, Gamma = 1	0.24(+/−0.66)	52.32(+/−18.63)	114.4(+/−25.81)	135.52(+/−17.00)
MUSCLE	FastME	JTT, Gamma = 1	0.16(+/−0.55)	36.96(+/−22.20)	105.84(+/−22.31)	132.88(+/−23.38)
MUSCLE	PhyML(Maximum-Liklihood)	LG	0(+/−0)	29.24(+/−25.77)	70.85(+/−21.73)	89.14(+/−24.14)
MUSCLE	PhyML(Maximum-Liklihood)	LG(+F)	0(+/−0)	38(+/−24.77)	75.28(+/−22.23)	96.08(+/−19.33)
MUSCLE	RAxML (Maximum-Liklihood)	WAG(+G)	0(+/−0)	7.76(+/−7.53)	43.28(+/−12.03)	63.2(+/−17.98)
MUSCLE	GARLI(Maximum-Liklihood)	WAG(+F)	0(+/−0)	8.8(+/−7.46)	46.16(+/−12.62)	65.68(+/−16.19)
CLUSTAL	Neighbor-Joining		NA	NA	NA	94.95(+/−10.05)
TCOFFEE	Neighbor-Joining		NA	NA	NA	149.42(+/−14.17)
MAFFT-L-INS-i	Neighbor-Joining		NA	NA	NA	153.52(+/−37.44)
MAFFT	Neighbor-Joining		NA	NA	NA	174.09(+/−2.05)
PHYRN	Neighbor-Joining		1.2(+/−1.53)	1.52(+/−1.66)	7.04(+/−4.05)	13.6(+/−4.12)
MUSCLE*	GARLI	WAG(+F), 10 inpendent runs	NA	NA	NA	61.27(+/−18.24)
PHYRN*	Neighbor-Joining		NA	NA	NA	14.6(+/−4.46)

# Performance described as RF distance +/−SD.

* Analysis done only on Data Sets 2 for ROSE 700.

### Methods for Estimating MSA and Phylogenetic Trees

We utilized a variety of MSA-based methods for each simulated data set. For a given data set, we obtained a MSA using MUSCLE v3.6 [22], DIALIGN v2.2.1 [23], MAFFT v6.833b [24], CLUSTALW v 2.0.12 [25], or T-COFFEE v 8.93 [26] with default parameters. Phylogenetic trees based on these MSAs were inferred by both distance- and character-based programs. For the distance-based condition, trees were inferred using the popular Neighbor-Joining (NJ) method and/or FastME methods. Further, we also explored more complex substitution models with character-based methods. Specifically, we tested various Maximum Likelihood (ML) algorithms for tree inference. PhyML v3.0 [27] was used at its default settings (using BioNJ to obtain the initial tree, and the Le and Gascuel (LG) amino acid replacement matrix [28]). Equilibrium amino acid frequencies were estimated from the data set using the +F option. RaxML v 7.0.4 parallel Pthreads version [29] is a different ML algorithm, and was used with the Whelan and Goldman (WAG) amino acid substitution model, and CAT approximations. CAT approximations were used in RaxML as it decreases computational time while retaining accuracy of tree inference. GARLI v 1.0 [30] (www.bio.utexas.edu/faculty/antisense/garli/Garli.html) was also used assuming the WAG amino acid substitution model and with the substitution frequencies estimated from the data in hand (+F settings). The gamma model of among-site rate variation was employed with empirical estimates of the extent of rate variation. In additional runs, we also used the recent version GARLI v2.0 [30]. To infer maximum parsimony trees we used both the PROTPARS program in the PHYLIP v3.67 package (http://evolution.genetics.washington.edu/phylip.html) and PAUP* (version 4) [31]. In data sets where parsimony method outputs multiple trees, only the best tree (based on RF distance) was used for average accuracy calculations. Finally, we tested the Bayesian method available in MrBayes 3.1.2 [32], incorporating its default settings with a mixed amino acid substitution model and a gamma model of among-site rate variation (and in additional runs using a gamma model of rate variation with a proportion of invariable amino acid sites). Default settings in MrBayes employ two different runs with 4 different chains between the 2 independent runs. Besides these default settings, we also utilized a parallel version of MrBayes with following settings: i) 16 parallel runs with the WAG amino sustitution substitution model and gamma model of among–site rate varaition, and ii) 32 parallel runs with the WAG amino acid substituition model. Optimal trees were obtained from two independent runs for each data set, and runs were stopped when runs reached stationarity (based on standard deviation of split frequencies, and also by examining the log likelihood values during runs). Majority-rule consensus trees after discarding first 25% samples as ‘burn-in’ were used for RF distance calculation. For each data set, consensus tree from the settings that provided best results was used in average RF distance calculations. For alignment-free methods, Average Common Substring(ACS) length-based distance [33] and Lempel-Ziv(LZ) distance [34] were calculated using ‘decaf+py’ package [35] , followed by tree inference using ‘neighbor’ program of PHYLIP package. All the settings and implementations used have been summarized in Table 1, and more details on commands is provided in Supporting Methods S1.

### Framework for PHYRN-Based Tree Inference

**Figure 1:**
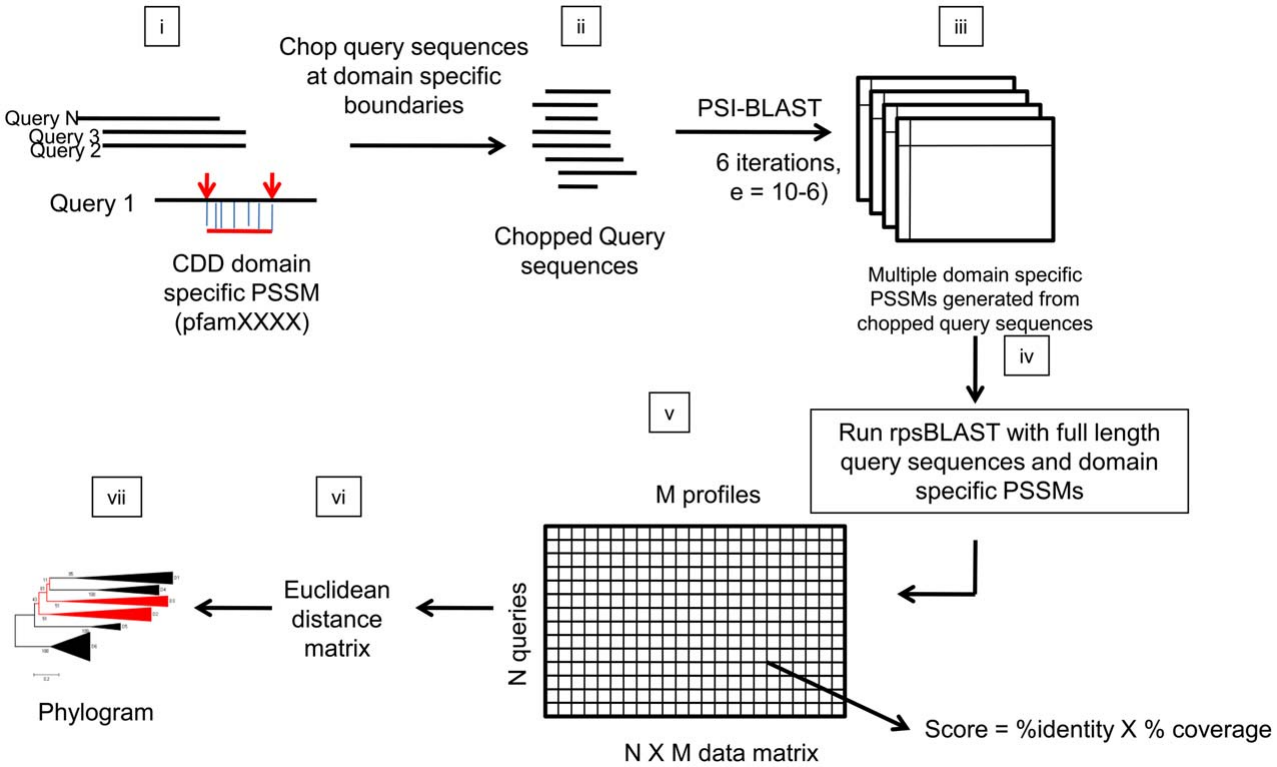
PHYRN concept and work flow. PHYRN begins by (i–ii) defining and extracting the domain specific region among the query sequences. (iii) Domain specific regions are then used to create PSSM library using PSI-BLAST. (iv–v) Positive alignments are then calculated between queries and PSSM library using rpsBLAST, and encoded as a PHYRN product score (percentage identity X percentage coverage) matrix. (vi) The product score matrix is converted to a Euclidean distance matrix by calculating Euclidean distance between each query pair. (vii) Phylogenetic trees are then inferred using Neighbor Joining, WEIGHBOR, Minimum Evolution, NINJA, or FastME.

The pipeline for the PHYRN algorithm is graphically represented in Figure 1. The input is a set N of amino acid sequences and set M of their associated PSSMs. The output is a tree *T*, leaf-labeled by the set N. In this study we tested four different tree building algorithms from our PHYRN distance matrix, including NJ, Weighbor [36] (weighted NJ), FastME [37] and NINJA [38]. PHYRN is a five step procedure: (i) curate a data set of amino acid sequences, (ii) construct a database/library of query-based PSSMs using PSI-BLAST, (iii) collect alignment statistics as a function of percentage identity X percentage coverage using a custom code of rps-BLAST and populate the real numbers into a NXM matrix, (iv) calculate Euclidean distance of all sequence pairs and represent distance in a NXN matrix, and (v) generate a distance-based tree estimated using Neighbor-Joining (or a similar clustering technique).

To generate PSSM libraries for the synthetic data sets, we used full-length sequences. Further, since there are no biological homologs for these synthetic sequences in the NCBI non-redundant (nr) database, full length sequences from synthetic data sets were added to the nr database, and PSSMs were generated from this modified non-redundant (nr) database. We used full-length synthetic sequences to generate PSSMs using PSI-BLAST with the aforementioned, modified nr database, at 6 iterations and e-value = 10^−6^. In contrast to our previous report [8], in this study we modified the product score to omit hits, excluded sequence embedding, and modified the PSSM library architecture to allow for increased computational speed in simulated libraries. Instead of organizing PSSM library as an assembly of individual single-domain databases, we changed library organization to have one single database comprised of all the PSSMs. In later sections on DANGER superfamily we illustrate how homologous regions from biological protein families can be identified and converted to PSSM libraries. Briefly this can be accomplished using several approaches such as (i) CDD profiles, (ii) an iterative use of PHYRN methodology, and/or (iii) sequence embedding based approaches [39], [40], [41]. All the codes and the user manual for PHYRN can be downloaded from www.ccp.psu.edu/downloads.

PHYRN uses a custom code for rps-BLAST for recording positive alignments between simulated sequences and their respective PSSM library. For a given profile M, the matrix is populated 0 for no alignment or as a positive product score for the alignment with best PHYRN score (%identity X %coverage) retrieved with an e-value threshold of 10^10^. Equation sets for calculating % identity and % coverage were defined as in previous studies [8]. However, unlike the composite score mentioned in Chang et al. [8] which included hits, in this case the PHYRN product score equals %identity X % coverage for each PSSM that provided an alignment [10]. Percentage identity (%i) and percentage coverage (%c) is defined as follows:

**%i** = [(Number of Identical residues in alignment)/(Alignment length including gaps)]

**%c** = [(Alignment length in query excluding gaps)/(Sequence length of PSSM)]

Thus, the PHYRN product score is directly proportional to the similarity between query sequence and PSSM, and inversely proportional to the gaps in an alignment. Overall, PHYRN product score provides a measurement of the length, robustness, and strength of the alignment. Mathematical derivations show that this PHYRN product score is equivalent to [(1-(Alignment restricted p-distance))*(1-PHYRN gap-weight)] (Equations i–v).

### Derivation of PHYRN Product Score

PHYRN product score  =  %Identity × %Coverage
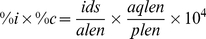
(i)where:

ids  =  number of identical residues in aligned region

alen =  length of the alignment

aqlen  =  length of the alignment without gaps

plen  =  length of the PSSM
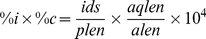
(ii)

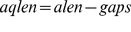
(iii)

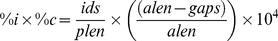
(iv)

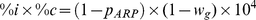
(v)


Alignment Restricted p-distance (p_ARP_) is defined as the proportion of amino acid sites different in alignment defined as a function of PSSM length. It is calculated by dividing number of non-identical amino acid sites by total length of the PSSM. PHYRN Gap Weight (w_g_) is defined as proportion of gaps defined as a function of alignment length. It is calculated by dividing total number of gaps in alignment by length of alignment.

From the NXM matrix, PHYRN calculates the Euclidian distance between each query [8] (Equation vi), which can then be depicted as a phylogenetic tree using a variety of tree-building algorithms.

Euclidean distance between two sequences *X* and *Y*, say *D(X, Y)*, is as follows:
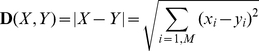
(vi)where X sequence is encoded as a vector of M scores (x_1_, x_2_, …, x_M_).

## Results

### Comparison of Tree Accuracy in Simulated Evolutions

**Figure 2:**
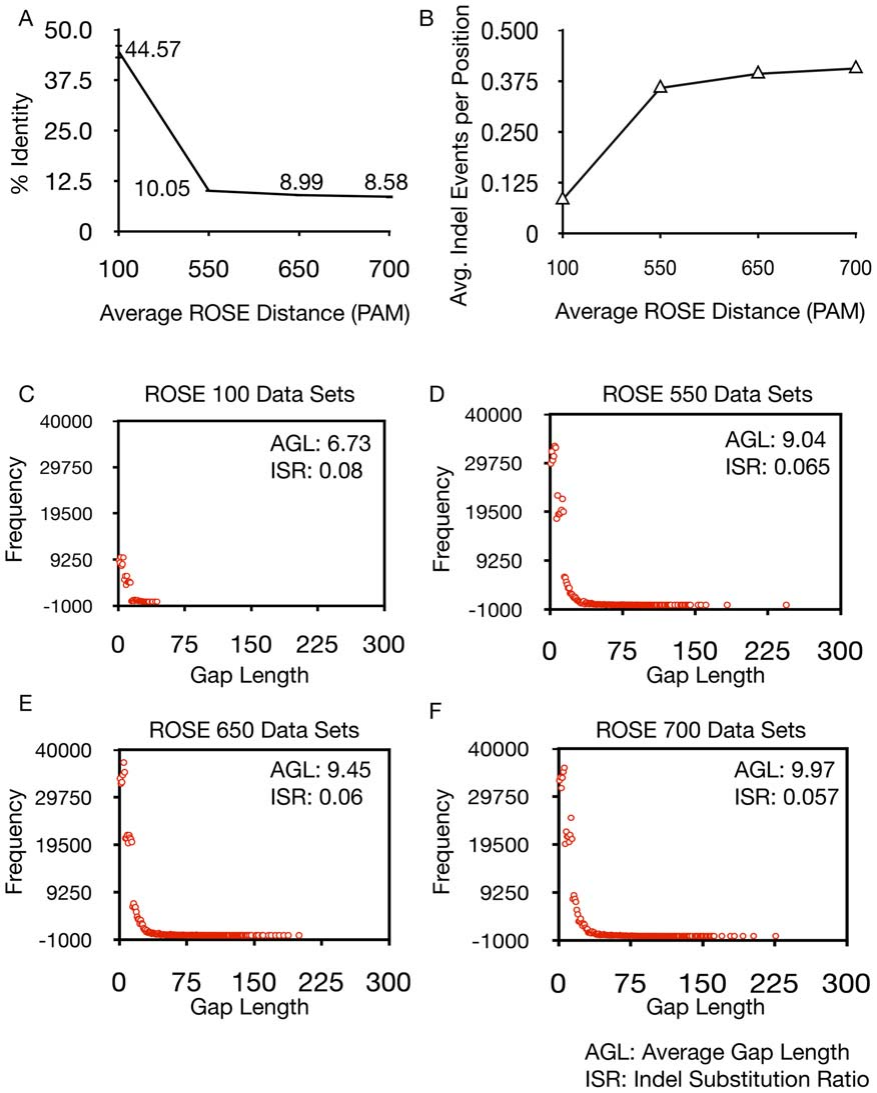
Characteristics of ROSE Data Sets. Multiple data sets (n = 25) were generated using ROSE at each divergence range (PAM distance  = 100–700). The true alignment provided by the ROSE simulation was used to calculate the percentage identity, and gap statistics. A) Average percent identity calculated from each data set, decreases on increasing PAM distance (n = 25, Error Bars: +/– S.E.M.). B) Distribution of Average INDEL Events per position at different divergence ranges (PAM100–700). Average Indel events are calculated by dividing total number of gaps by total number of amino acid positions in all sequences represented in 25 replicates. C−F) Distribution of gap lengths in all replicates generated at PAM 100-PAM700. (Number of replicates = 25, number of sequences in each replicate  =  100. Average length of each sequence = 450 aa). AGL: Average Gap Length as calculated from the mean of all gap lengths in all 25 replicates. ISR: Indel event Rate/Substitution Rate.

Figure 2A–F depicts how the percentage identity and gap statistics change between PAM 100 and PAM 700 of ROSE generated data sets. We observe that for trees constructed at an overall distance of PAM 550, average percentage identity as calculated from true alignments provided by ROSE is as low as 10% (Fig 2A). In data sets generated at PAM 650 and PAM 700, the average percent identity of data sets falls to 8.99% and 8.58%, respectively. We also observe that indel substitution events (i.e. gap openings) calculated as a function of each amino acid position, also increase with increasing PAM distance (Fig 2B). Moreover, we plotted the frequency distribution of all the gaps in 25 replicates at each divergence range (Number of data sets at each range = 25, Number of sequences in each data set = 100). We observe that with increasing PAM distance, average gap length (AGL) and the frequency of gaps increases (Fig 2C–F); however, the ratio of indel rate to substitution rate (ISR) does not change significantly between PAM 550 and PAM 700. In summary, our comparative statistics across PAM distances demonstrate that increasing PAM distance increases: 1) substitution rates, 2) frequency of gap events, and 3) the average length of gaps.

**Figure 3:**
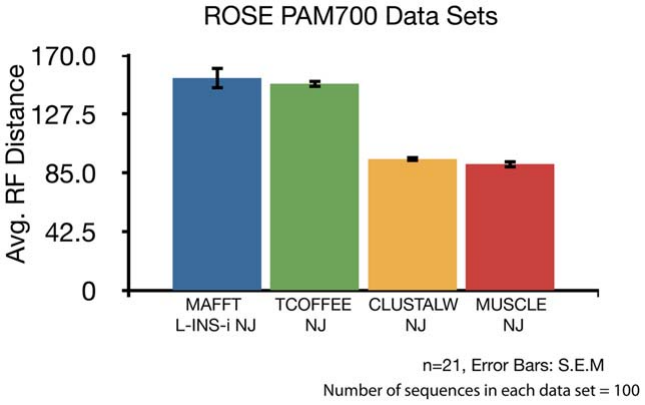
Accuracy Comparison of Different MSA methods. Graphical representation of average Robinson-Foulds Distance from true ROSE trees (n = 21, PAM700) generated using different MSA methods. All trees were inferred using Neighbor-Joining. (n = 21, Error Bars: +/− S. E. M.). The number of sequences in each data set = 100. Maximum possible RF distance = 194.

Using these data sets, we first determined the most accurate MSA method for benchmarking in our study. We tested the performance of multiple popular MSA methods in these data sets (MAFFT, MUSCLE, TCOFFEE, and CLUSTAL). We generated trees for 25 different ROSE data sets at an average distance of PAM 700. For rapid comparisons, we employed the NJ algorithm for these analyses. Since we employed a single tree-inference method, phylogenetic accuracy in this analysis is a function of MSA quality. Figure 3 shows that MUSCLE and CLUSTAL have improved performance over MAFFT and TCOFFEE that is statistically significant (p<0.01). However, MUSCLE and CLUSTAL have statistically similar performance. Therefore, for the rest of our study, we used MUSCLE as it is computationally much more efficient than CLUSTAL. In data not shown, we tested additional MSA methods (i.e. Dialign, and K-align); however, these were excluded from Figure 3, as they could not generate trees in data sets above PAM 550.

In Figure S1 we present our initial comparative analysis of PHYRN and MUSCLE-NJ using both the SeqGen and ROSE data sets generated at seven different ranges of divergence (40%–7% identity, Figure S2 C–D). In total, we performed 425 simulations totaling 1655 tree comparisons. At the lower levels of divergence, PHYRN marginally outperforms MUSCLE-NJ at recapitulating deep nodes; however, at higher divergences, PHYRN performs significantly better in both the SeqGen and ROSE data sets.

**Figure 4:**
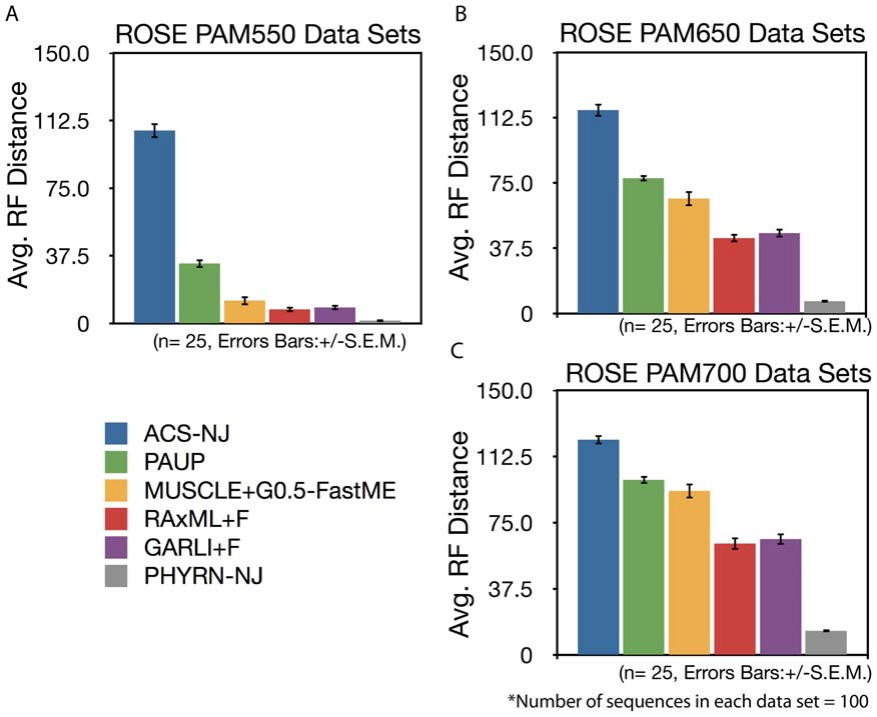
Performance Comparison of PHYRN with other Phylogenetic Inference methods. A–C) Graphical representation of average symmetric distance (Robinson-Foulds Distance) between the true ROSE tree and trees estimated using PHYRN, ACS-NJ (Average Common Substring, Alignment-free method), MUSCLE-FastME (corrected distance method), MSA-PAUP (Maximum Parsimony), MSA-RAxML (Maximum Likelihood), and MUSCLE-GARLI (Maximum Likelihood) based methods. Number of replicates tested at each divergence range = 25, Error bars = +/− S.E.M. The number of sequences in each data set = 100, Avg. Length of sequences = 450. Maximum possible RF Distance = 194.

To extend upon our previous comparative analysis we next benchmarked against alignment-free, maximum parsimony, corrected distance, and ML methods (Figure 4 and Table 1). For this analysis, simulated data sets were generated at four different PAM distances (PAM 100, 550, 650, and 700). For each divergence range we generated 25 different data sets comprised of 100 sequences each with an average sequence length of 450 amino acids. All ML analyses were conducted with the substitution frequencies estimated from the data to ensure the best performance of these algorithms (+F option). Substitution matrix and other parameters used for each method are listed in Table 1.

At the lowest level of divergence (PAM 100), all methods perform equally well (Table 1). In addition, for all data sets, we observe that alignment-free, FastME and Maximum Parsimony (MP) perform poorly when compared to RaxML and GARLI, while RaxML and GARLI perform similarly. However, in our PAM 550 data set (25 replicates, ∼ 10% identity), RaxML and GARLI have an average RF-distance of 7.76 and 8.8, respectively, while PHYRN has an average RF-distance of 1.52, which is significantly lower than all methods tested (p<0.0001). Similarly, in our PAM 650 (25 replicates, ∼8.9% identity), RaxML and GARLI have average RF-distances of 43.6 and 46.16 respectively, while PHYRN remains robust with an average RF-distance of 7.04. In the most divergent data set we tested in this experiment, PAM 700 (25 replicates, ∼8.5% identity), RaxML and GARLI have average RF-distances of 63.84 and 65.68, respectively. Once again, PHYRN remains relatively robust with an average RF-distance of 13.6. We also tested multiple other methods and settings (NJ, corrected vs. uncorrected distances, Lempel-Ziv distance, PhyML, MP using Protpars, variations in substitutions matrices, gamma rate (+G), and empirical frequencies (+F)), the results of which are shown in Table 1. Overall, we observe that at high rates of sequence divergence, PHYRN provides statistically more accurate inference of tree topologies than other methods (and their implementations) tested here. To test whether other distance-based tree-inference methods besides NJ would improve PHYRN performance, we tried WEIGHBOR, Ninja, and FASTME (Fig S2). Importantly, the performance of PHYRN is consistent regardless of the tree-building method employed. Since all methods tested here produced similar results, we suggest that the PHYRN distances derived are robust.

**Figure 5:**
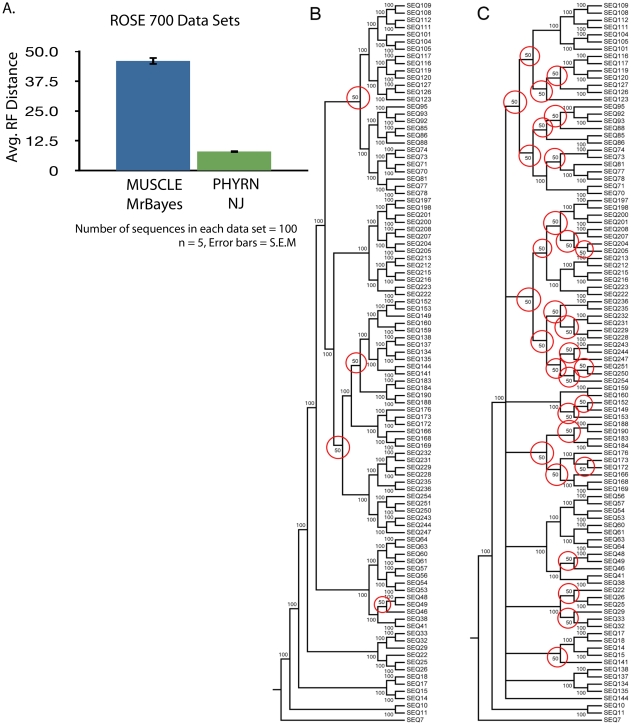
PHYRN outperforms MrBayes in ‘midnight-zone’ synthetic data sets. A) Graphical representation of symmetric distance (from true ROSE trees) for trees inferred using PHYRN and MrBayes. Data sets used were generated using ROSE at PAM 700 distance. Number of data sets tested (n = 5). The number of sequences in each data set = 100. Maximum possible RF distance = 194. Error Bars: +/−S.E.M. B) Consensus tree between true ROSE tree and PHYRN tree (PAM 700 data set 1). Red circles mark nodes that are incorrectly inferred by PHYRN. C) Consensus tree between true ROSE tree and MrBayes tree (PAM 700 data set 1). Red circles mark nodes that are incorrectly inferred by MrBayes.

As a final comparison of PHYRN performance, we compared it with the Bayesian method MrBayes [32]. Since this Bayesian approach is extremely computationally expensive we compared only five data sets at PAM 700. In this analysis, PHYRN consistently yielded a lower RF-distance, and thus more phylogenetic accuracy, than MrBayes (Figure 5A). Specifically, MUSCLE-MrBAYES has an average RF-distance of 46.0, while PHYRN has an RF-distance of 8.0 for these five data sets. The differences in performance are highlighted in Figure 5B,C. Panels 5B depict a consensus tree between one trial of PHYRN versus the True ROSE tree; a branch value of 100 means that PHYRN inferred the correct branching pattern while a value of 50 means that PHYRN incorrectly inferred that branch. Panel 5C depicts the analogous results from one trial of MrBayes versus the True ROSE tree. From this we observe that PHYRN has only four branching errors, while MrBayes contains 30.

Following these results, we examined the scalability of PHYRN with a single data set comprised of 1000 sequences and a mean distance of PAM550. Consensus tree between the true ROSE tree and the PHYRN-NJ tree shows that PHYRN infers only 8 branches incorrectly out of total 1998 branches. Moreover, the PHYRN-NJ tree shows a symmetric RF distance of 14 to the true ROSE tree (Figure S3). In data not shown we also tested the efficacy of PHYRN using different tree topologies at extreme divergences. In both biased (i.e. unbalanced) and unbiased trees (i.e. balanced), PHYRN outperforms all MSA-based methods analyzed here. However, in highly biased trees, all methods fail to perform due to the extreme divergence that occurs at the basal nodes. Thus, additional experimentation is needed to resolve highly biased evolutionary histories.

### Isolating Variables Underlying Phylogenetic Accuracy

**Figure 6:**
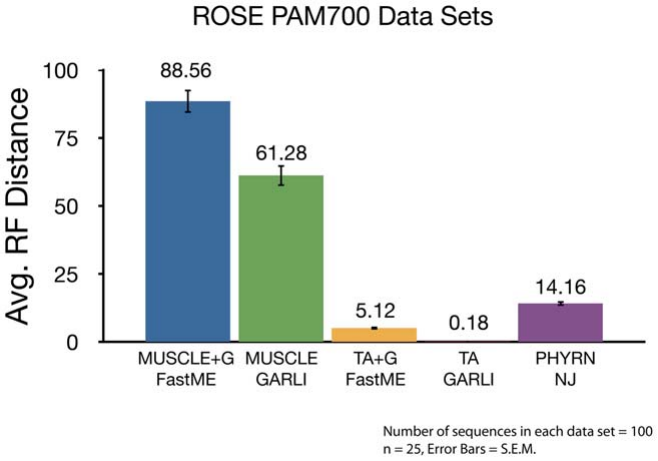
Effect of ‘True Alignment’ on Phylogenetic Inference. Graphical representation of average symmetric distance (Robinson-Foulds Distance) between the true ROSE tree and trees estimated using PHYRN, corrected distance (FastME) and ML methods (GARLI). Corrected Distance and ML trees were generated with both MUSCLE alignment, and True Alignment (TA) provided by ROSE. (Number of replicates tested at each divergence range = 25, Error bars = +/− S.E.M. Number of sequences in each data set = 100, Avg. Length of sequences = 450). Maximum possible RF distance = 194.

The relatively poor performance we observe for MSA-based methods in this study could be due to either sub-optimal MSA quality and/or inaccurate tree inference. To discriminate between these variables we employed the true-alignments as provided by ROSE. If phylogenetic accuracy is not substantially improved, we can infer that the tree-inference method used is sub-optimal. In this experiment, we used the 25 data sets generated at PAM 700 distance from ROSE, and trees were inferred using corrected FastME, and GARLI. Notably, trees inferred using the true alignment perform very well, with an average RF distance of 5.12 using FastME, and 0.18 using GARLI (Figure 6). Hence, these data demonstrate that poor phylogenetic accuracy as observed in earlier comparisons is largely due to poor MSA quality. Indeed, Figure 6 shows that when these same data sets are aligned by MUSCLE, both FastME and GARLI markedly lose phylogenetic accuracy. Since PHYRN does not use an MSA step, we could not use the true alignment with this method, but PHYRN using its default methodology gives an average RF distance of 14.16. In sum, these results show that phylogenetic inference in divergent data sets is stymied by the sub-optimal quality of MSA, not by tree-inference methods.

### DANGER Superfamily as a Phylogenetic Test-Bed

To determine the efficacy of PHYRN in a biologically relevant data set, we examine sequences from the highly divergent DANGER superfamily. This developmental superfamily is ubiquitously expressed and is linked to multiple physiological (Ca^2+^ signaling, cranio-facial development, reproduction, neurite outgrowth [42], [43], [44]) and pathophysiological (excitotoxicity) processes [45]. Until recently, the MAB21-domain containing superfamily DANGER escaped detection due to their extreme divergence [42]. Indeed, significant genetic correlates were required to support monophyletic groups for this superfamily. A previous study by our group, which relied on MSA-based approaches, defined six distinct monophyletic groups of DANGER [42]. Although the orthologous relationships were well defined, the paralogous relationships in this family were ambiguous. Indeed, even upon rigorous genetic analyses and extensive manual editing of these alignments, deep-node statistical support was unattainable.

### Implementation of PHYRN for Biological Data

In the simulated data sets reported earlier, we utilized the full-length sequences to generate PSSMs. However, biological data sets are often comprised of both homologous and non-homologous regions. Previous studies have demonstrated that phylogenetic inference in divergent data sets improves when measurements of phylogenetic signal are limited to homologous regions [3], [10]. Therefore, we sought to further refine PSSM generation by limiting the PHYRN-based measurement of phylogenetic signal to homologous regions and to generate PSSM libraries for these areas of interest (see Supporting Methods S1 for complete description). Briefly, we curated protein sequences belonging to the DANGER superfamily from the literature and sequence databases. All known DANGER members (D1–D6 groups) share the MAB-21 domain in common [42]. Therefore, we aligned each putative DANGER member against PSSMs for the MAB-21 domain as defined by NCBI Conserved Domain Database (CDD) [46]. These alignments were utilized to define the homologous region in each protein sequence. Together, these regions were converted to a MAB21-specific PSSM library containing 112 PSSMs using PSI-BLAST and compiled into an rpsBLAST compatible database.

Once an appropriate PSSM library is constructed, the next step is to align all queries with all PSSMs and encode the alignment statistics into an N×M matrix. In this format, N is the number of full-length query sequences and M is the number of PSSMs in the library. Hence, we aligned 108 full-length DANGER query sequences against 112 Mab-21 PSSMs using rpsBLAST. A composite score matrix (%identity×%coverage) was generated by encoding alignment statistics for all query-PSSM alignments. The pairwise distances among them (i.e. N×N matrix) were based on Euclidean distance measurement in the 108×112 data matrix. Finally, we inferred a phylogenetic tree from this matrix with the Neighbor-Joining (NJ) method available in the MEGA package [47] (See Supporting Methods S1 for more details.).

### Comparative Analyses of Inferred Trees

**Figure 7:**
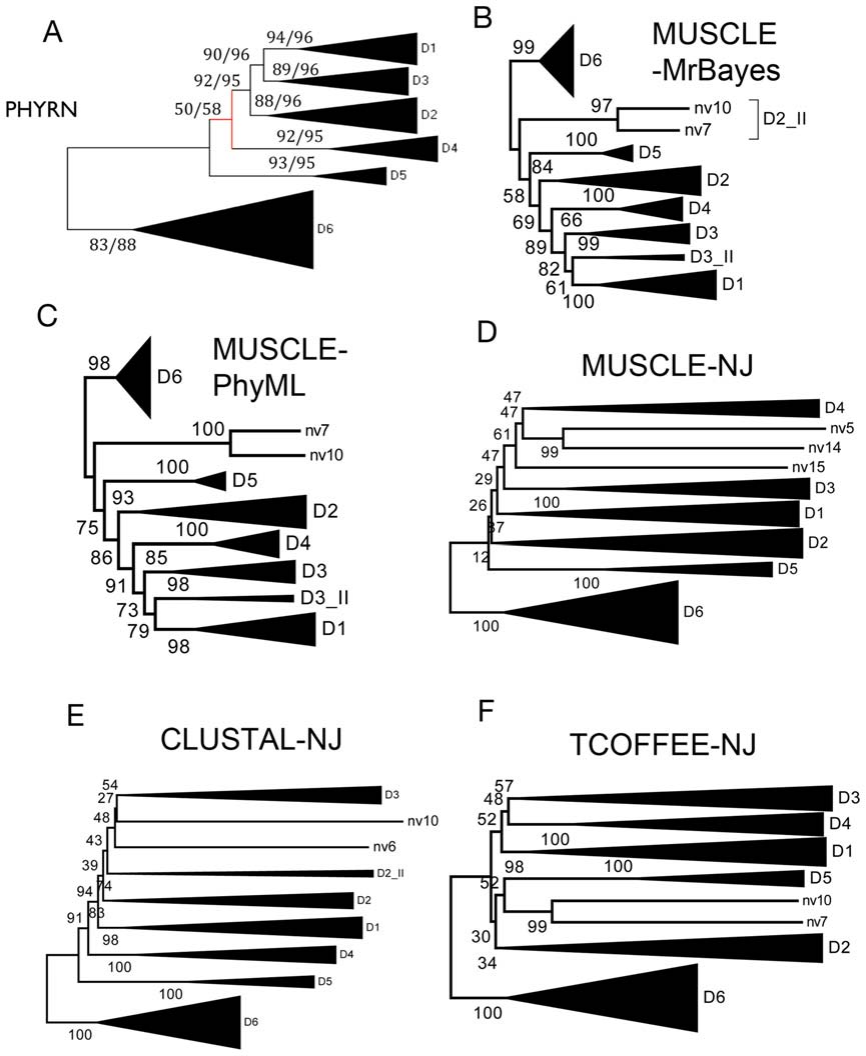
Comparison of Topology and Resampling Statistics for Various Tree Construction Methods. Collapsed unrooted phylogenetic trees for DANGER superfamily generated using (A) PHYRN-NJ, (B) MUSCLE-MrBayes, (C) MUSCLE-PhyML, (D) MUSCLE-NJ, (E) CLUSTAL-NJ and (F) TCOFFEE-NJ. For PHYRN trees the statistics are represented by two numbers with Bootstrap listed first followed by Jacknife statistics. Statistics for panel A were calculated from resampling results from 3000 replicates. Bootstrap statistics for panels B-F were calculated from resampling results from 1000 replicates.

To compare PHYRN-based trees with traditional methods, we generated phylogenetic trees with the same DANGER sequences using a variety of MSA and tree building algorithms. These include: (i) MUSCLE-MrBayes [5], [22], [32], (ii) MUSCLE-PhyML [5], [22], [27], (iii) CLUSTAL-NJ [25], and (iv) TCOFFEE-NJ [26] (Figure 6). Together these five approaches are representative of traditional character-based and distance-based methods for phylogenetic inference and are a good subset of methods for comparative analysis with PHRYN. Figure 7a and Figure S4a depict the unrooted tree derived by PHYRN, from which we observe plausible biological patterning of six monophyletic groups (D1–D6) in accord with our previous studies. [42]. For example, within the D6 clade, cnidaria (e.g. sea anemone) occupies a basal position, followed by nematode (e.g. worms), urochordates (e.g. sea squirt), arthropods (e.g. insects), and chordates (e.g. humans). However, a single sequence from sea urchin diverges subsequent to arthropods, and thus appears to be misplaced. Trees generated by MUSCLE-NJ, MUSCLE-PhyML, CLUSTAL-NJ, and TCOFFEE-NJ also place this sequence in the same, possibly erroneous position (Figure 7b–e). By comparison, MUSCLE-MrBayes lacks monophyly for various groups, such as members of D2 and D3 clades and incorrectly places *Nematostella* (i.e. Cnidaria- sea anemone) D3 sequences after other higher order organisms. CLUSTAL-NJ tree splits members of D2 clade, and places some *Nematostella* sequences after the mammalian specific group D1. MUSCLE-NJ and TCOFFEE-NJ trees also misplace *Nematostella* sequences. MUSCLE-PhyML provides good bootstrap support but splits members of D3 clade. Thus, all methods with the exception of PHYRN either fail to infer monophyly, and/or yield a tree with an improbable evolutionary scenario.

To assess the statistical support for these various phylogenetic trees we conducted an 80% jack-knife resampling for PHRYN and bootstrap resampling for all approaches. By these measures PHYRN obtains support of >83% (bootstrap) and >88% (jack-knife) for all deep-nodes except for the placement of the D4 clade. Conversely, none of the other traditional methods tested obtain significant results for any deep-node other than the D5/D6 clades which is the most conserved subgroup in the superfamily. Figure S4 depicts the unrooted and non-collapsed phylogenetic trees for PHYRN and MrBayes with resampling statistics at all branch points. On leaf nodes, both methods perform equally well; however, there are major differences between the topology and branch statistics between methods. Overall, this suggests that PHYRN has increased ability to measure low phylogenetic signal.

### Meta-Analysis of PHYRN-Derived Data

In our previous evolutionary study of DANGER, we identified a single sequence from choanoflagellate, which was used as the putative outgroup [42]. Importantly, this sequence obtained no statistical support for this position. To ascertain whether this sequence was indeed an outgroup, we searched for additional putative DANGER sequences in multiple publically available sequence databases including NCBI, Community Cyberinfrastructure for Advanced Marine Microbial Ecology Research and Analysis (CAMERA, [48]), and Department of Energy Joint Genome Institute (JGI) databases. Taken together, we identified 13 additional *Monosiga* sequences (i.e. Choanoflagellate- microscopic, heterotrophic single-celled and colony-forming eukaryotes). When we incorporate these sequences into our analyses, PHYRN infers a monophyletic topology; however, the choanoflagellate sequences form a distinct clade, with D3 as the nearest neighbor (Figure S5a). Moreover, their inclusion drastically reduces the statistical support across the entire tree (compare Figure 7a and Figure S5a). Based upon this observation, the homology of these *Monosiga* sequences with the DANGER superfamily is highly questionable and is likely in error.

From the matrix data generated by PHYRN, we can obtain additional quantitative measurements such as group-wise distribution of composite scores of sequence to PSSM comparisons, as well as their information content. These measures can be utilized to interrogate placement of the *Monosiga* group in the DANGER phylogeny. Figure S5b demonstrates that in all cases, these choanoflagellate PSSMs have the fewest alignments across all clades, and their sequences have the lowest information content (average product score, ±S.E.M). Moreover, the positions of the choanoflagellate sequences relative to the vertebrate specific D1 clade within the tree are suspect. In this scenario, multiple clades that contain ancient species (e.g. cnidarians, nematodes, and arthropods) would have evolved after D1. Thus, in order for this scenario to make sense, D1 proteins would have to be lost from all species prior to chordates, which is not parsimonious. Final evidence that these choanoflagellate sequences are not homologous to DANGER and thus do not belong in the phylogeny come from exhaustive searches of sequence databases. We could not identify any DANGER sequences in species before choanoflagellate or between choanoflagellate and cnidaria.

**Figure 8:**
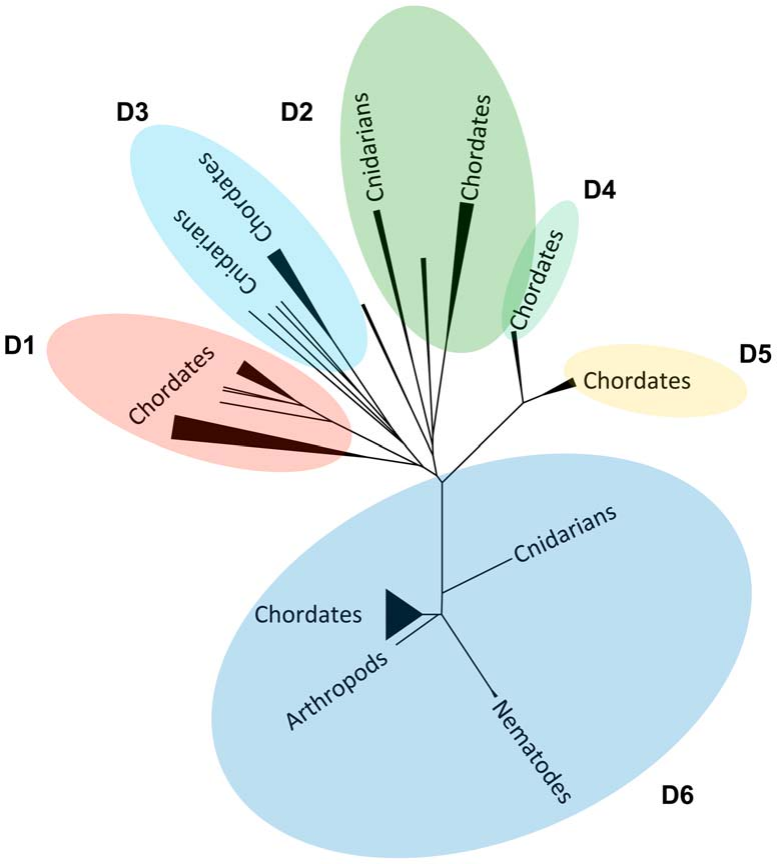
Model for the Evolution of the DANGER Superfamily. Graphical representation of the Neighbor-Joining (NJ) tree for 108 DANGER sequences generated PHYRN. In this model, DANGER appeared first in cnidarian organisms *(Nematostella)* and then evolved into 6 different clades. The chordate specific group, D1 attains the furthest position from the putative root (D6).

Thus, the question arises: which DANGER clade is the oldest? In our quantitative statistics, we observe that PSSMs from the D6 clade have the highest group-wise distribution and D6 sequences have the highest information content (Figure S5b). Further, in the unrooted tree D6 clade has the longest branch-length. Taken together, D6 is the most logical outgroup of the superfamily based on (i) statistical support, (ii) information content, and (iii) speciation. Taken together, our results suggest the following evolutionary scenario (Figure 8). The first DANGER sequences emerged in cnidarians (>580 million years ago), which are some of the first organisms known to have a developed neural net, radial axis of symmetry, muscle cells, and stem cells [49], [50]. Accordingly, members of the DANGER superfamily have been shown by functional studies to be involved in neurite length extension [42], calcium mobilization [43], and developmental patterning [44], [51], [52]. If we root our phylogenetic tree with D6 (Figure 8), we see a “simple to complex” evolutionary pattern for the DANGER superfamily, with the mammalian-specific D1 clade attaining the most distant position. Similarly, we see the appearance of simpler organisms before more complex organisms within individual monophyletic groups. For example, in D6 clade, cnidarians are the first ones to show DANGER followed by nematodes, arthropods and then chordates. Importantly, we could not identify cnidarian sequences in D4 and D5 clades. This is relevant because relatively newer clades D3 and D2 do have cnidarian members, and suggests that D4 and D5 were lost from cnidaria.

## Discussion

Within divergent biological data sets it is impossible to know the true evolutionary history of sequences under consideration. Due to the lack of knowledge about true evolutionary history, there is no way to accurately evaluate the performance of algorithms on biological data sets. Therefore, in the present study we utilized simulations as test beds of phylogenetic inference. Only in this way can one measure a true evolutionary history, and hence accurately quantify the performance of various algorithms by comparing ‘inferred history’ to ‘true history’. Indeed, synthetic data sets have frequently been used for benchmarking algorithm performance [12], [13], [14], [15], [16]. Our comparative analysis within synthetic protein and biological sequence data indicate that PHYRN can provide a more accurate and statistically robust representation of evolutionary history within the “twilight zone/midnight zone” of sequence similarity as compared to multiple popular MSA-approaches. Our interpretation is supported by several key findings, including; 1) PHYRN outperforms all distance and ML methods tested given a MUSCLE alignment, 2) PHYRN outperforms a Bayesian method (MrBayes), given a MUSCLE alignment, and 3) both, distance-based and character-based methods require the true alignment in order to outperform PHYRN.

While simulations do not entirely reflect all of the underlying mechanisms of natural molecular evolution, they still represent powerful approximations of the evolutionary process (for example, including substitution matrices derived from biological databases, inclusion of insertions-deletions) and we have tested our method on two of the most-utilized simulation methods (SeqGen and ROSE). While more research is needed to develop improved models of evolution that more accurately reflect biological mechanisms, this does not detract from their utility for benchmarking studies, and PHYRN also appeared to perform well on a real biological data set comprising a highly divergent superfamily of developmental proteins (the DANGER superfamily).

In both synthetic and biological data sets we reason that the improved performance of PHYRN is due to the increased information content contained in PSSM libraries and an effective alignment search and scoring method. Conversely, the inability of MSA methods to obtain accurate alignments at high divergence leads to low accuracy of trees across all tree-building methods. At the lower end of this performance spectrum Neighbor-Joining performed well in conserved data sets, but poorly at higher levels of divergence. Some ML methods (RAxML and GARLI) perform better than NJ, but their performance is also greatly limited by the quality of input MSA methods. Bayesian methods, which are computationally very slow (because a whole posterior distribution of trees are produced), show a similar performance to RAxML and GARLI. We also considered other approaches such as PROBCONS and other consistency based models, but these have been shown to be slower, and thus are not easily scalable. Moreover, PROBCONS has been previously benchmarked in the twilight-zone [53], and which showed that PROBCONS performs no better than ClustalX, Align-m, T-Coffee, SAGA, ProbCons, MAFFT, MUSCLE and DIALIGN. More generally, our results on tree inference using true ROSE alignments show that better alignments may be the key to estimating accurate phylogenies in highly divergent data sets (figure 6).

For those MSA-based methods tested, we have tried to give these algorithms the best opportunity to perform well. In addition to comparisons with the default settings, we also explored: (i) equilibrium frequencies estimated from the empirical data, and (ii) a variety of among-site rate variation models in the Bayesian method. Importantly, most of these settings did not improve performance to any great extent. An ideal MSA method at extreme divergence involves “cleaning” for badly aligned regions followed by tree-inference. To accomplish this goal, we filtered our MSAs using Gblocks [12]; strikingly, however, our simulated data sets are so divergent that Gblocks fails to recognize any conserved sequence blocks. Thus, in these simulated data sets it is impossible to simply filter out badly aligned regions. Nevertheless, we acknowledge that there are still settings that could be fine-tuned to improve alignment estimation and tree inference in a data set dependent manner. In particular, although we have tried to benchmark against many popular MSA methods, further experimentation is needed to benchmark PHYRN against other MSA algorithms such as Prank [54] and SATe [55]. In addition, we also need to explore PHYRN's performance in data sets where substitution rates deviate substantially from a molecular clock, and where evolutionary models are permitted to change across a phylogeny.

In conclusion, we propose that our increased performance on synthetic and biological data sets demonstrates that PHYRN is an accurate and scalable approach. We suggest that PHYRN's ability to handle large numbers of highly divergent sequences makes it an ideal framework to study a number of unanswered questions relating to some of the earliest events in the history of life. Future work will focus on exploring: (i) the utility of PHRYN-based ‘guide trees’ for improving MSA-based algorithms, (ii) the integration of PHYRN-based distance estimates with other statistical methods such as Maximum Likelihood, and (iii) the refinement of PHYRN-based PSSM libraries with Markovian statistics (i.e. HMM profiles) [56].

## Supporting Information

Figure S1 **PHYRN outperforms MSA in synthetic protein families.** Consensus tree between true ROSE tree and tree generated using a) PHYRN and b) MUSCLE with NJ. Simulated protein family generated using ROSE, with an average distance of 550 (p distance ∼0.83). Red circles mark the branch points (nodes) that are recapitulated incorrectly. (# of query sequences = 67). c) Graphical representation of %deep node recapitulation versus SeqGen scaling factor. Number of replicates for each bar = 25, Error bars  =  +/− S.E.M. *p-value<0.01. Number of sequences in each data set = 100, Length of sequences = 450. d) Graphical representation of %deep node recapitulation versus average Rose distance. Number of replicates for each bar = 25, Error bars = +/− S.E.M. *p-value < 0.01. Number of sequences in each data set = 100, Avg. Length of sequences = 450.
(PDF)

Figure S2 **Effect of Tree Inference Method on PHYRN Performance.** Graphical representation of symmetric distance for trees inferred from PHYRN distance matrix and different tree inference methods. Number of replicates tested at each divergence range = 25, Error bars = +/− S.E.M. Number of sequences in each data set = 100, Avg. Length of sequences = 450. (Maximum possible RF distance for each data set = 194).
(PDF)

Figure S3 **Deep node recapitulation of ‘true evolutionary history’ in mega-phylogenies.** Consensus phylogenetic tree between true ROSE tree and tree generated using PHYRN. The simulated protein family was generated using ROSE, with an average PAM distance of 550. (Red colored branches mark the branches that are recapitulated incorrectly in the consensus trees. (number of query sequences  =  1000). PHYRN recapitulates 1990 branches correctly out of total 1998 branches in the consensus tree. PHYRN shows a RF distance of 14 from the true ROSE tree (Maximum possible RF distance for this data set = 1994).
(PDF)

Figure S4 **Comparison of PHYRN and MrBayes generated Trees for DANGER Superfamily.** Unrooted Phylogenetic trees for 108 DANGER sequences generated using (A) PHYRN or (B) MUSCLE-MrBayes. Statistical support for PHYRN calculated using Bootstrap and Jackknife analysis, while for MUSCLE-MrBayes only bootstrap was used. The blank marked ‘‘_/_’’ in the statistical support indicates that the clustering of the branching connection cannot be measured in a standardized fashion by the given resampling method.
(PDF)

Figure S5 **Identification of most basal DANGER clade using PHYRN quantitative measures.** (A) DANGER tree generated by PHYRN-NJ including 13 *Monosiga* sequences. The tree is drawn to scale, with branch lengths in the same units as those of the Euclidean distances. Statistical support was calculated using Bootstrap and Jackknife analysis from 3,000 replicates and are reported as percentages with bootstrap values labeled first. (B) This bar graph depicts addition quantitative measures derived by PHYRN for group-wise distribution of composite score (i.e. percentage identity X percentage coverage). Errors bars = +/−S.E.M. In all cases, choanoflagellate sequences have the lowest information content (average PHYRN product score, ± S.E.M).
(PDF)

Supporting Methods S1 **Supplemental methods describing PHYRN PSSM generation and simulation parameters.**
(PDF)
