# Supplementary figures and images for: PHYRN: A Robust Method for Phylogenetic Analysis of Highly Divergent Sequences
Source: PLoS One. 2012 Apr 13;7(4):e34261. doi: 10.1371/journal.pone.0034261 (PMC3325999; doi:10.1371/journal.pone.0034261)

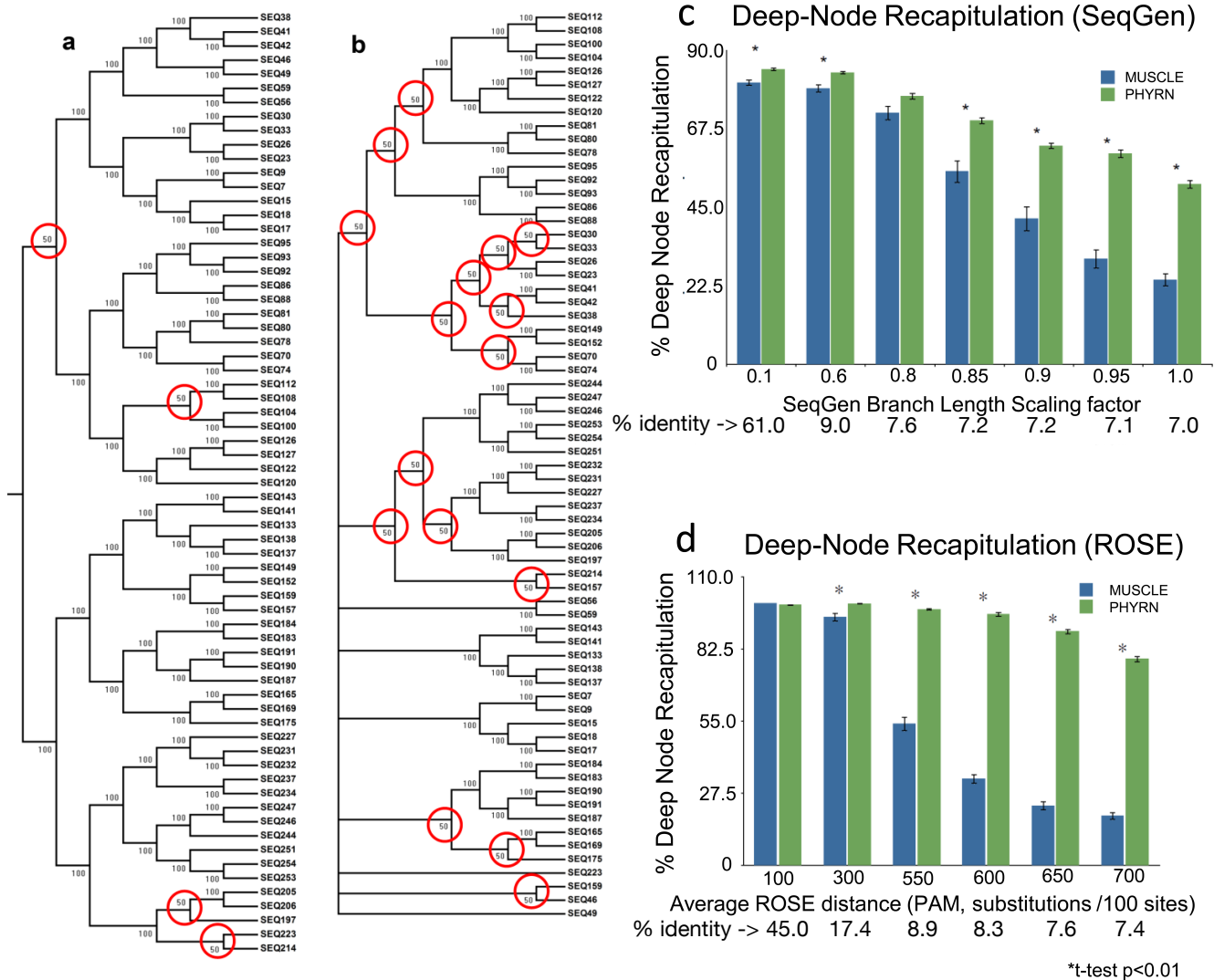

\*t-test  $p < 0.01$

Figure S1

Supplement: Figure S1 — PHYRN outperforms MSA in synthetic protein families. Consensus tree between true ROSE tree and tree generated using a) PHYRN and b) MUSCLE with NJ. Simulated protein family generated using ROSE, with an average distance of 550 (p distance ∼0.83). Red circles mark the branch points (nodes) that are recapitulated incorrectly. (# of query sequences = 67). c) Graphical representation of %deep node recapitulation versus SeqGen scaling factor. Number of replicates for each bar = 25, Error bars = +/− S.E.M. *p-value<0.01. Number of sequences in each data set = 100, Length of sequences = 450. d) Graphical representation of %deep node recapitulation versus average Rose distance. Number of replicates for each bar = 25, Error bars = +/− S.E.M. *p-value < 0.01. Number of sequences in each data set = 100, Avg. Length of sequences = 450. (PDF) [file pone.0034261.s001.pdf]

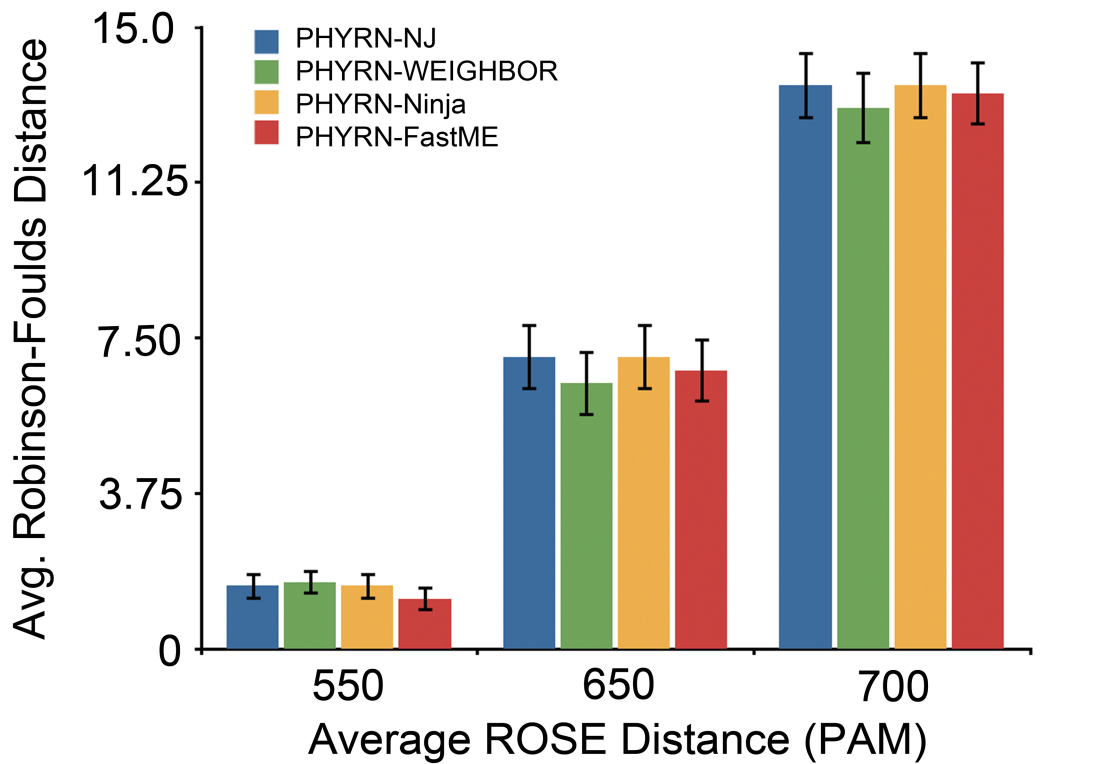

Figure S2

n=25, Error Bars=S.E.M.

Supplement: Figure S2 — Effect of Tree Inference Method on PHYRN Performance. Graphical representation of symmetric distance for trees inferred from PHYRN distance matrix and different tree inference methods. Number of replicates tested at each divergence range = 25, Error bars = +/− S.E.M. Number of sequences in each data set = 100, Avg. Length of sequences = 450. (Maximum possible RF distance for each data set = 194). (PDF) [file pone.0034261.s002.pdf]

ROSE 550 Data Set  
Number of Sequences = 1000

PHYRN-NJ

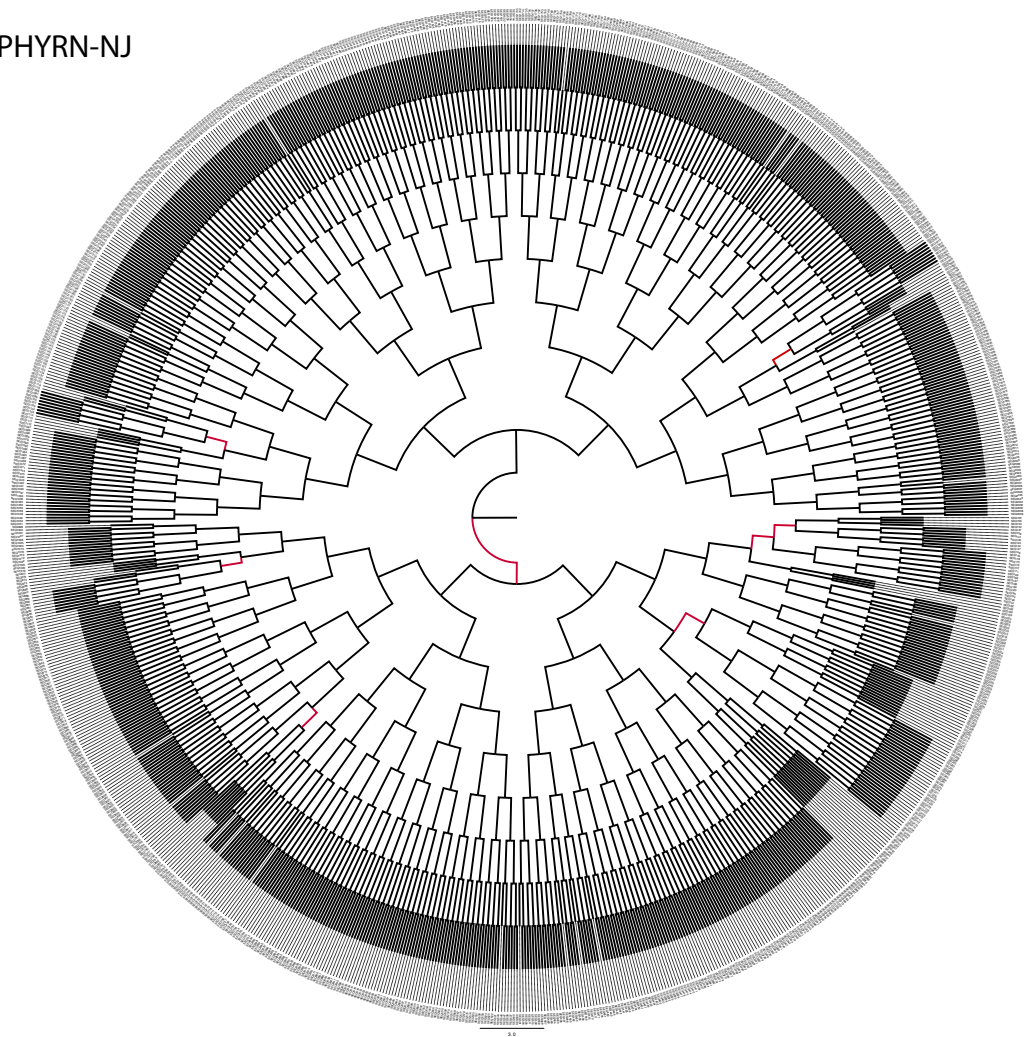

Red: Incorrect Branches

Figure S3

Supplement: Figure S3 — Deep node recapitulation of ‘true evolutionary history’ in mega-phylogenies. Consensus phylogenetic tree between true ROSE tree and tree generated using PHYRN. The simulated protein family was generated using ROSE, with an average PAM distance of 550. (Red colored branches mark the branches that are recapitulated incorrectly in the consensus trees. (number of query sequences = 1000). PHYRN recapitulates 1990 branches correctly out of total 1998 branches in the consensus tree. PHYRN shows a RF distance of 14 from the true ROSE tree (Maximum possible RF distance for this data set = 1994). (PDF) [file pone.0034261.s003.pdf]

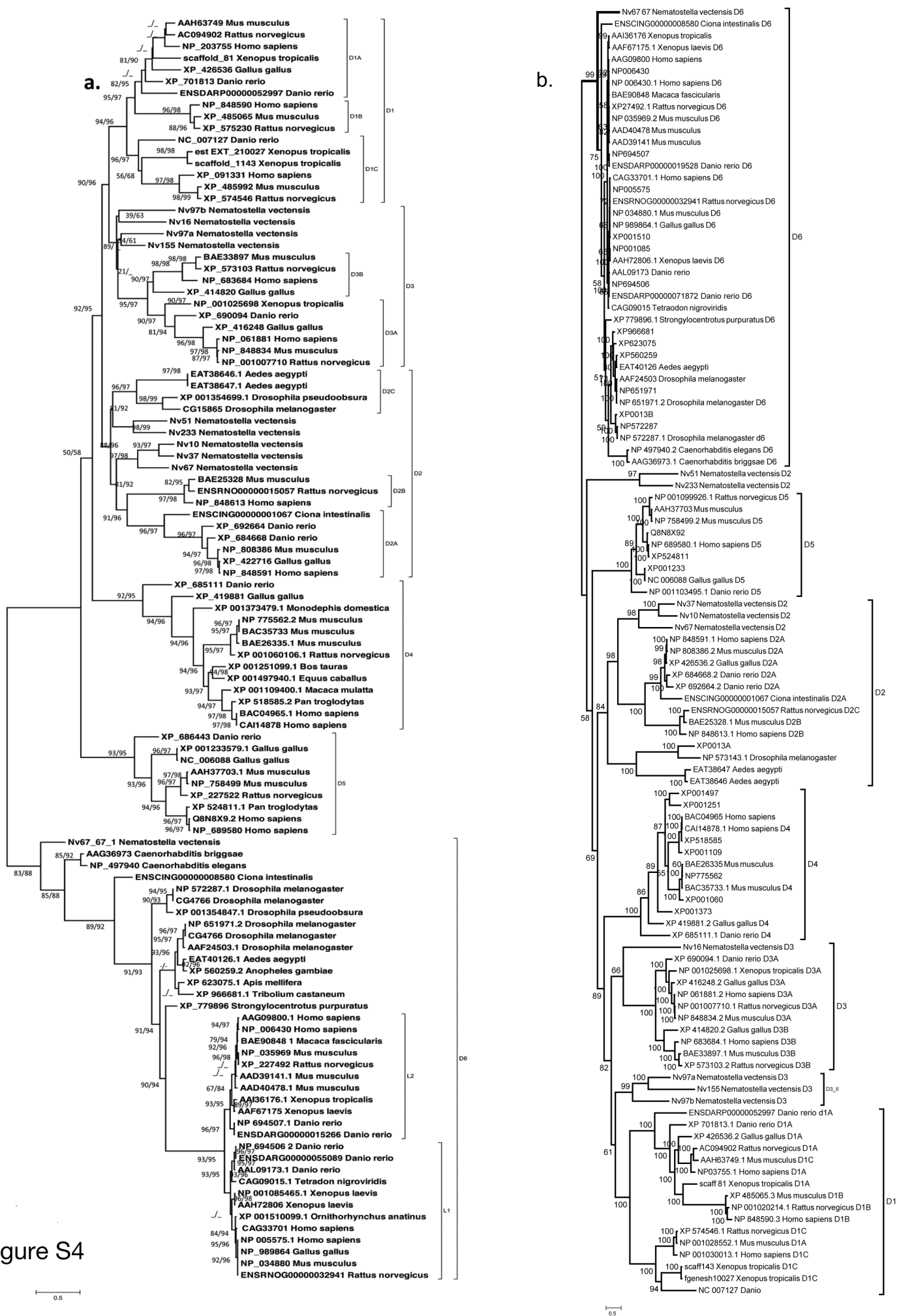

Figure S4

Supplement: Figure S4 — Comparison of PHYRN and MrBayes generated Trees for DANGER Superfamily. Unrooted Phylogenetic trees for 108 DANGER sequences generated using (A) PHYRN or (B) MUSCLE-MrBayes. Statistical support for PHYRN calculated using Bootstrap and Jackknife analysis, while for MUSCLE-MrBayes only bootstrap was used. The blank marked ‘‘_/_’’ in the statistical support indicates that the clustering of the branching connection cannot be measured in a standardized fashion by the given resampling method. (PDF) [file pone.0034261.s004.pdf]

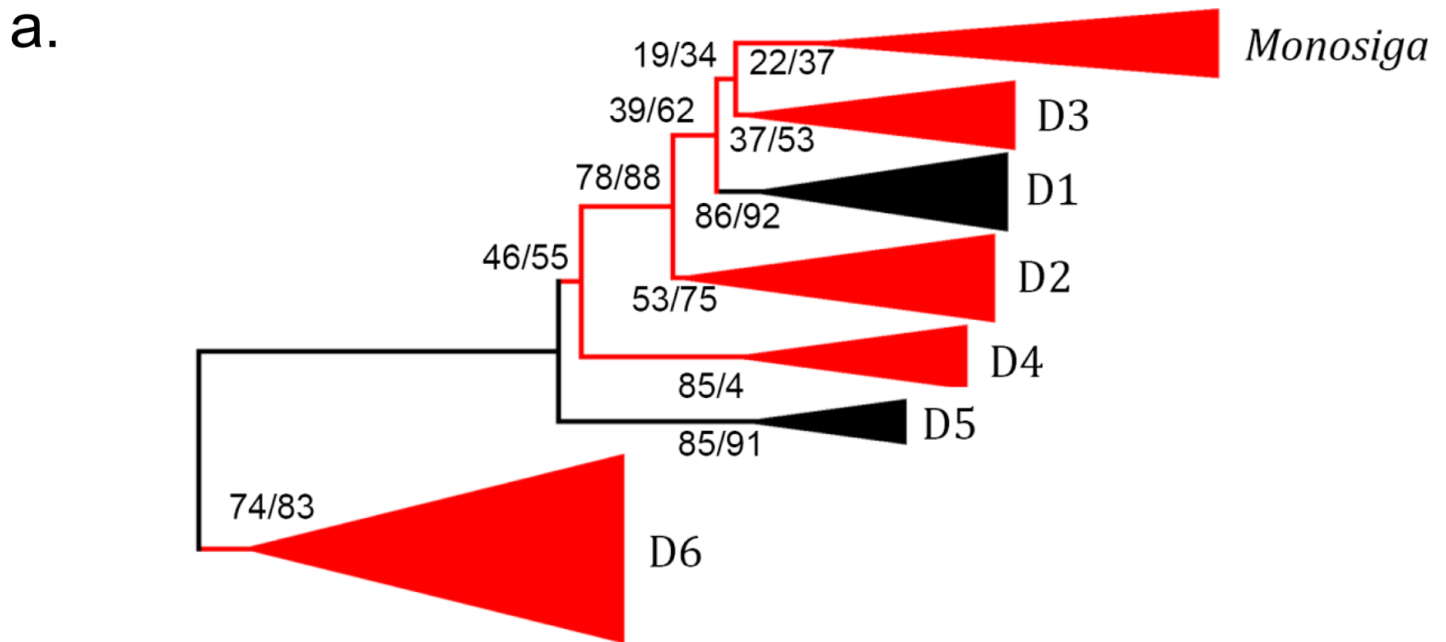

b.

Group-Wise PHYRN score (%i X %c) Distribution

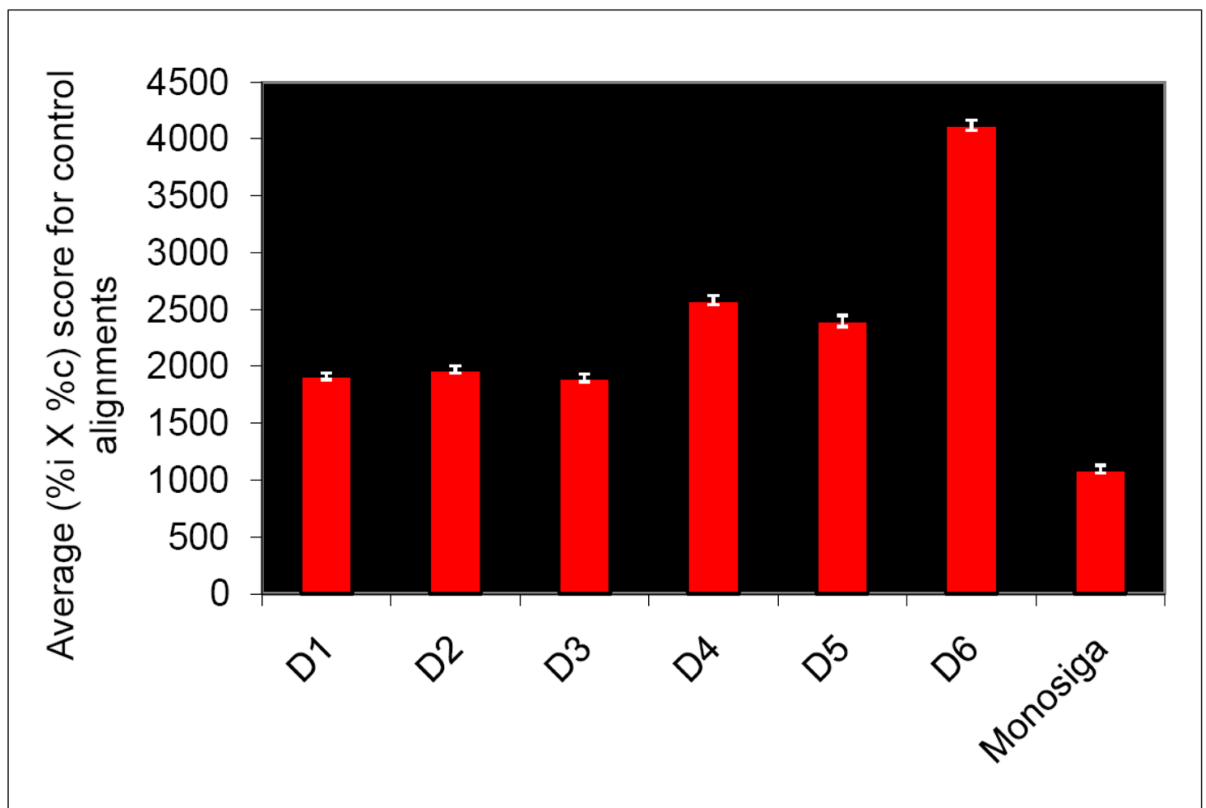

Supplement: Figure S5 — Identification of most basal DANGER clade using PHYRN quantitative measures. (A) DANGER tree generated by PHYRN-NJ including 13 Monosiga sequences. The tree is drawn to scale, with branch lengths in the same units as those of the Euclidean distances. Statistical support was calculated using Bootstrap and Jackknife analysis from 3,000 replicates and are reported as percentages with bootstrap values labeled first. (B) This bar graph depicts addition quantitative measures derived by PHYRN for group-wise distribution of composite score (i.e. percentage identity X percentage coverage). Errors bars = +/−S.E.M. In all cases, choanoflagellate sequences have the lowest information content (average PHYRN product score, ± S.E.M). (PDF) [file pone.0034261.s005.pdf]
