# Supplementary material for: PHYRN: A Robust Method for Phylogenetic Analysis of Highly Divergent Sequences
Source: PLoS One. 2012 Apr 13;7(4):e34261. doi: 10.1371/journal.pone.0034261 (PMC3325999; doi:10.1371/journal.pone.0034261)
Supplement: Supporting Methods S1 — Supplemental methods describing PHYRN PSSM generation and simulation parameters. (PDF) [file pone.0034261.s006.pdf]

Supplemental Methods

1. PHYRN methodology

Our PHYRN algorithm begins by compiling a set of protein queries belonging to same protein family (Figure 1). We used the Conserved Domain Database (CDD) to define conserved domains present in members of a superfamily (e.g. the Mab21 domain in DANGER). From this subset of knowledgebase PSSMs we utilize pairwise comparisons to define boundaries of sequence homology. These homologous protein fragments are then utilized to construct a database/library of query-based PSSMs using PSI-BLAST (6-iterations, e-value threshold= 10-6) Generating PSSM library from homologous regions reduces noise from non-homologous regions in phylogenetic measurements, and effectively samples the variability in homologous points during PSSM generation. In this manner, a given query set can be represented as a large library of PSSMs. In the case of the synthetic data sets, simulated sequences were inserted into the non-redundant protein database to accurately simulate the PSSM generation of biological sequences (see Methods for full description). We then use rpsBLAST to obtain pairwise alignments between full-length queries and the query-specific PSSM library. Alignment information is encoded into phylogenetic profile matrix as a PHYRN product score (percentage identity X percentage coverage). Percent identity (% i) and percent coverage (%c) are defined as follows:

%i = {(Number of Identical residues in alignment) / (Alignment length including gaps)}

%c = {(Alignment length in query excluding gaps) / (Sequence length of PSSM)}

Thus PHYRN product score is directly proportional to the similarity between query sequence and PSSM, and inversely proportional to the gaps in an alignment. Also PHYRN product score provides a measurement of the length of the alignment, as well as the strength of the alignment. Mathematical derivations show that this PHYRN product score is equivalent to [(1-(p-distance))*(1-gap-weight)] (see Equation 5 in manuscript). Next, we calculate the Euclidian distance between each query, which can then be depicted as a phylogenetic tree using a variety of tree-building algorithms (e.g. Neighbor-Joining, Minimum Evolution). In following sections, we demonstrate how to generate a three-node tree for DANGER from three full-length (FL) sequences and Mab-21 specific PSSMs. (also see following figures)

Step1: Identification and Extraction of Homologous regions

Query sequences belonging to the same protein family/super-family are collected from literature and/or sequence databases (e.g. Query Sequence1-3 from DANGER superfamily). Query Sequences are run against NCBI CDD to identify homologous regions among the query sequence set. In our data set, all queries have a Mab-21 superfamily domain. Further, Homologous regions thus identified are extracted from full-length queries using a custom python script (available at www.ccp.psu.edu/downloads)

Step2: Construction of a homologous PSSM library

Homologous regions extracted in step1 are used as fasta sequences for creating PSSM library using PSI-BLAST at settings of 6 iterations and 1e-6 e-value threshold. Since each query contributes at least one PSSM, the number of PSSMs (m) is equal to number of queries (n). In cases where some queries have multiple conserved homologous regions, we select all the regions for PSSM generation. All the PSSMs thus generated are compiled as single PSSM library. (e.g. Mab-21 specific PSSM library). We used blastpgp and formatrpsdb programs from BLASTALL package to convert homologous regions into PSSM libraries. Commands used for construction libraries as part of PHYRN pipeline are as follows:

```
rpsblast -d <nr_database> -i <input file> -o <output_file>
-C <matrix outfile> -e 0.01 -m 0 -s T -a 1 -h 1.0e-6 -j 6 -
M BLOSUM62 -F F -u 1 -J T
```

Individual PSSMs are compiled into a single library using following command.

```
formatrpsdb -i <pssm file list> -o T -n <library name>
```

Step3: Collecting and Scoring alignments between Queries and PSSM Library

Full-length query sequences were searched for alignments with the PSSM library (generated in step2) using rpsBLAST at e=1010 threshold. This extremely relaxed threshold collects all possible alignments, and lets us select for the best alignment based on its identity and coverage values. rpsblast prom BLASTALL package was used with following command.

```
rpsblast -i <query file> -d <PSSM Library> -e 1e10 -m 8 -o
<output file>
```

Step4: Encoding Alignments as evolutionary distance vectors

All alignments between Query and PSSM pairs are encoded into an n X m matrix of %identity X %coverage scores, where n is the number of queries and m is the number of PSSMs. Euclidean distance is calculated between each query pair from this composite score n X m matrix. Thus, the n X m composite score matrix is converted into a n X n euclidean distance matrix. This euclidean distance matrix can the be used to generate a phylogenetic tree using Neighbor-joining (NJ), Minimum evolution (ME) algorithms.

Step1: Identification and Extraction of Homologous regions

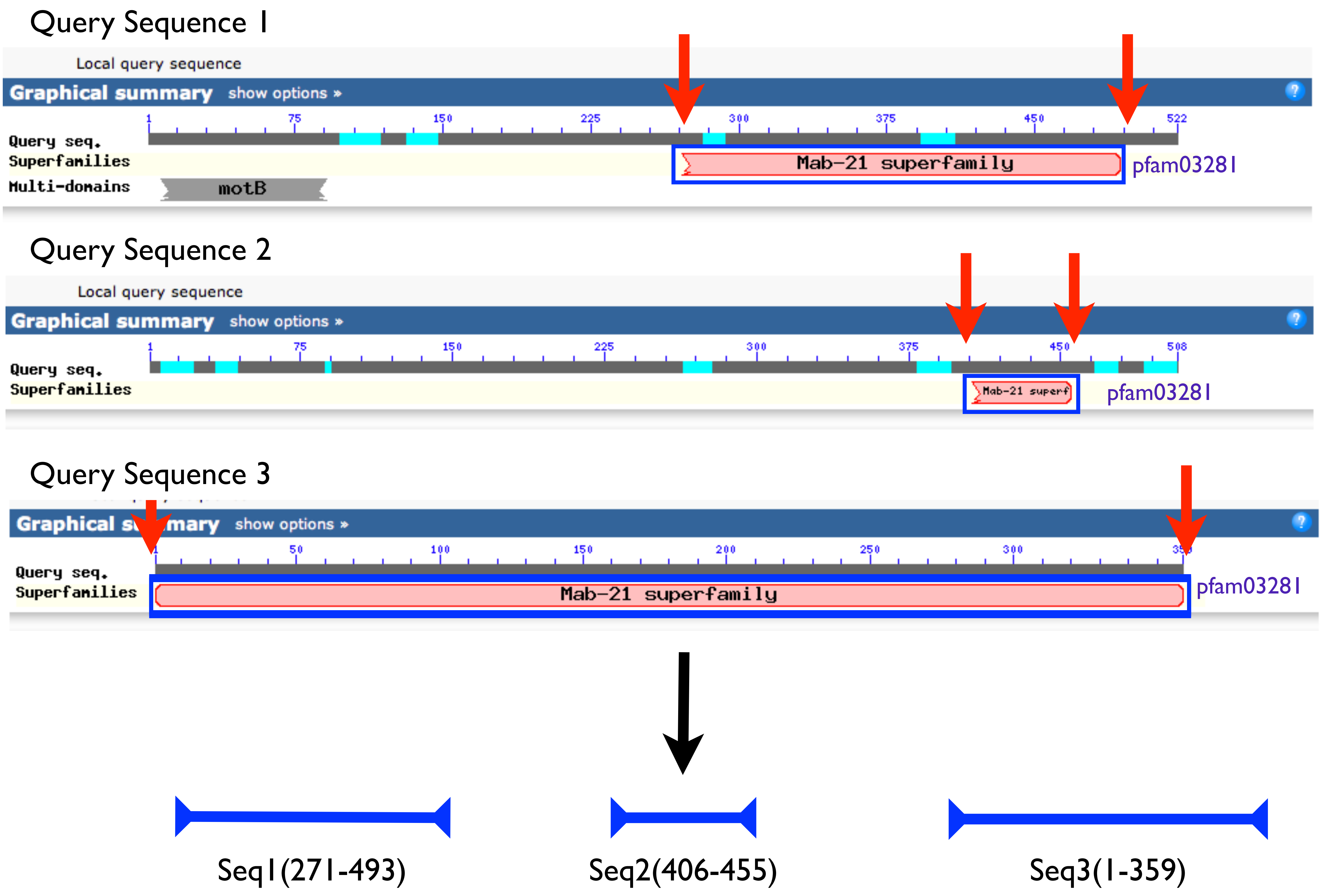

Step 2: Construction of a homologous PSSM library

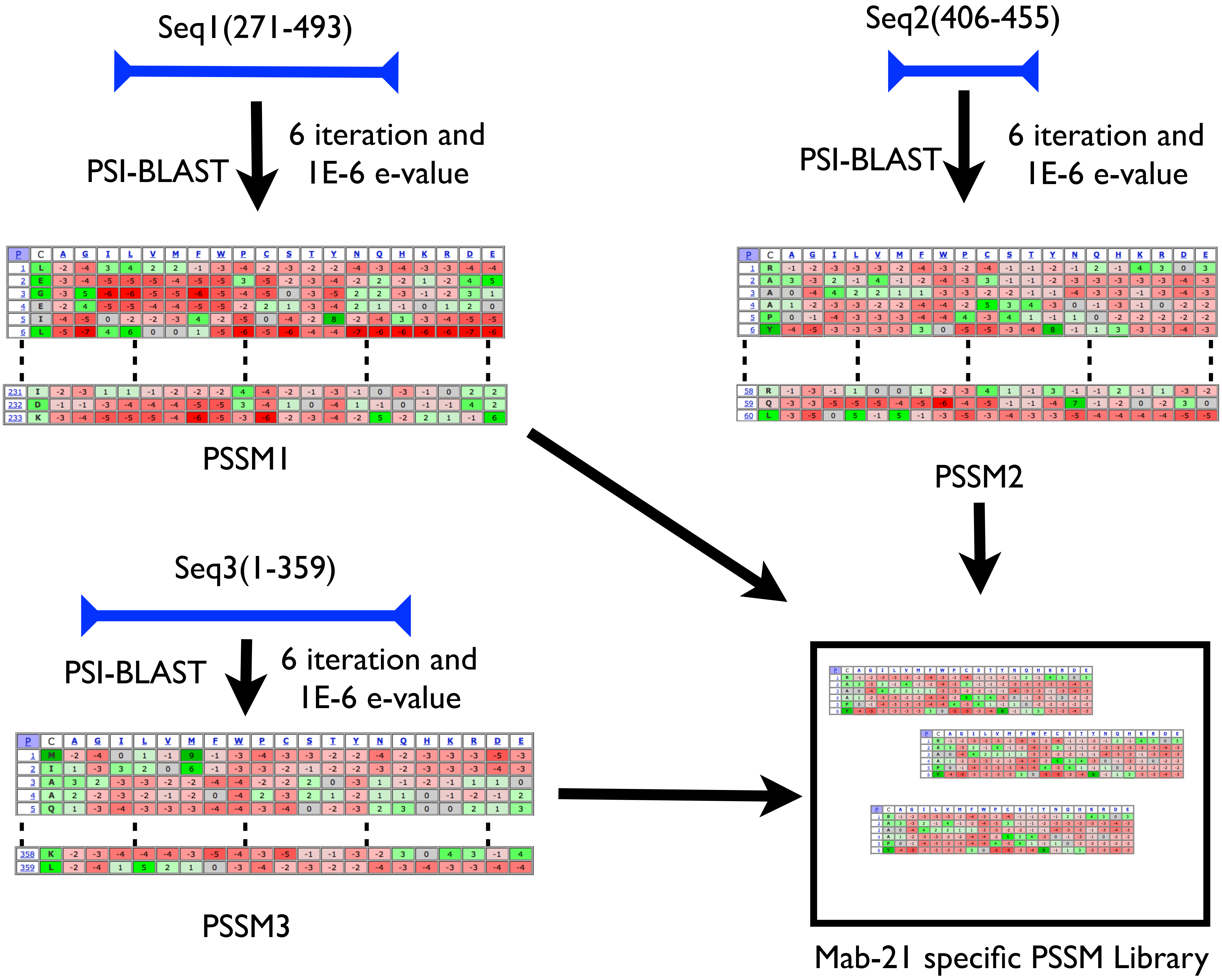

Step 3: Collecting and Scoring Alignments between Queries and PSSM library

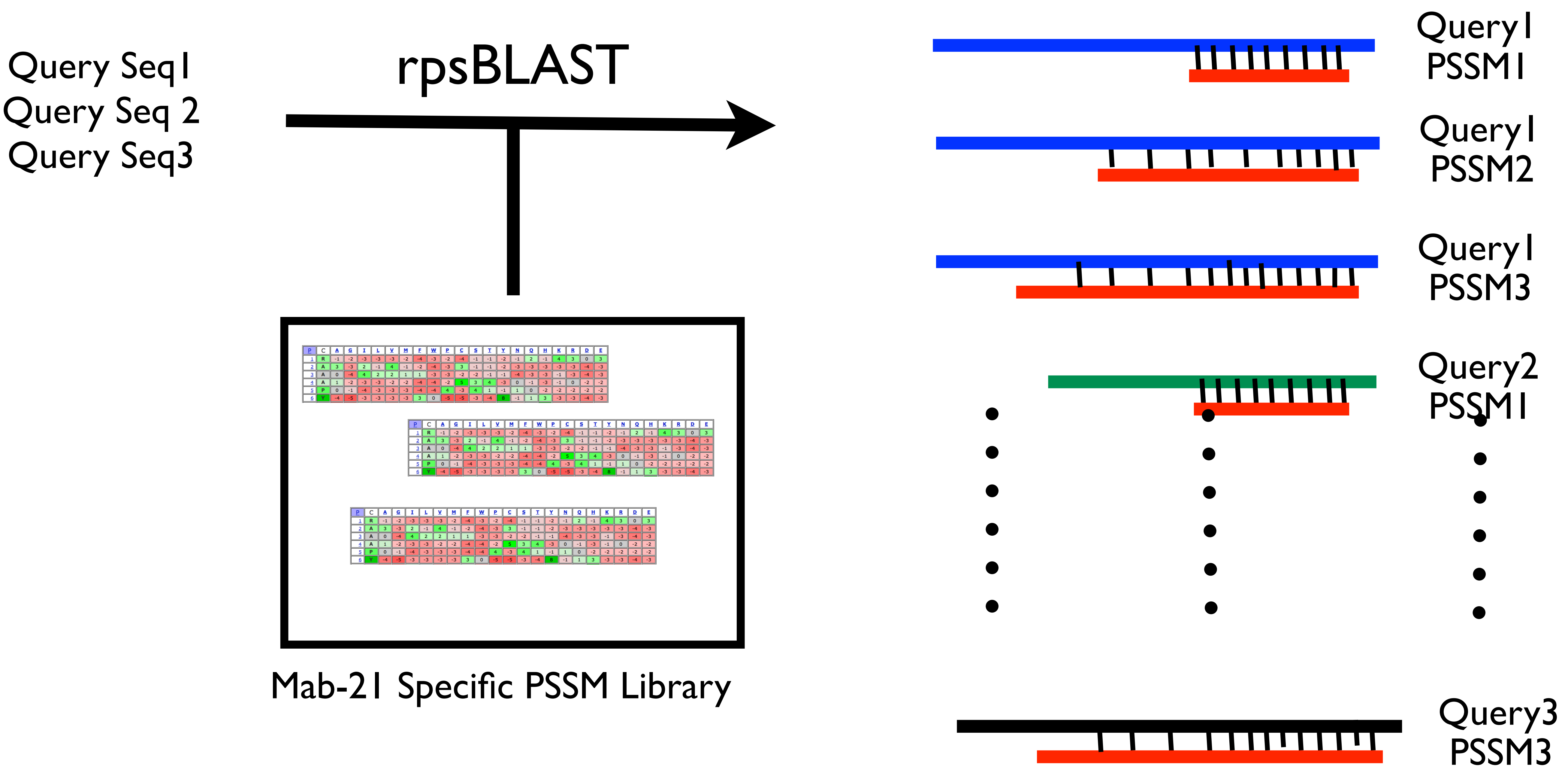

% Identity of alignment (i) = (Number of identical residues)/(Alignment Length including gaps)

% Identity of alignment (i) ∝ Strength of alignment

% coverage of alignment (c) = (Alignment length without gaps)/(Sequence length of PSSM)

% coverage of alignment (i) ∝ Robustness of alignment

Composite Product Score (cp) = %identity X %coverage

Step4: Encoding alignments as evolutionary distance vectors

	PSSM 1	PSSM 2	PSSM 3
Query 1	CP_{11}	CP_{12}	CP_{13}
Query 2	CP_{21}	CP_{22}	CP_{23}
Query 3	CP_{31}	CP_{32}	CP_{33}

CP (Composite Score) = %identity X %coverage

→ Euclidean distance

	Query 1	Query 2	Query 3
Query 1	ED_{11}	ED_{12}	ED_{13}
Query 2	ED_{21}	ED_{22}	ED_{23}
Query 3	ED_{31}	ED_{32}	ED_{33}

Euclidean Distance matrix

Neighbor Joining (NJ)/Minimum Evolution (ME)

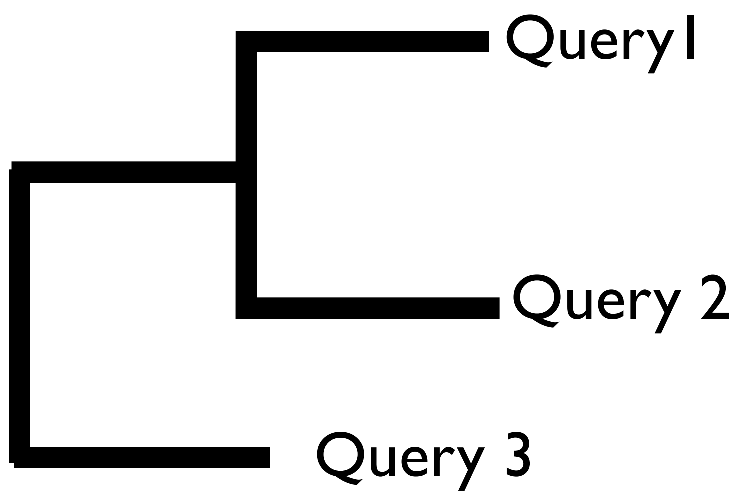

2. ALIGNMENT GENERATION AND TREE INFERENCE

2.1MULTIPLE SEQUENCE ALIGNMENT (MSA)

Multiple Sequence alignments was generated using MUSCLE v3.6, CLUSTALW v 2.0.12, TCOFFEE v 8.93, and MAFFT v6.833b

MUSCLE-Default

```
muscle -in <input file> -out <output file>
```

CLUSTALW-Default

```
clustalw2 -align -INFILE=<input file>  -output=pir -
OUTFILE=<output file>
```

T-COFFEE Default

```
t_coffee_Version_8.93 <input file>  -output <output file>
```

MAFFT-Default

```
mafft <input file>  > <output file>
```

MAFFT-L-insi

```
Mafft-linsi <input file> > <output file>
```

2.2 ALIGNMENT FREE METHODS

Average Common Substring (ACS) distance and Lempel-Ziv (LZ) distance algorithm as implemented in decaf+py package were implemented with default settings. Distance Matrices thus obtained were used to calculate Neighbor-joining trees using NEIGHBOR program from PHYLIP v. 3.67 with ‘L’ setting for lower order matrix.

```
python compute-acs-distance.py -f <input file> -o <output
matrix>
```

```
python compute-lz-distance.py –f <input file> -o <output
matrix>
```

2.3 DISTANCE METHODS

NEIGHBOR program from PHYLIP v. 3.67 was used with default settings to calculate Neighbor-Joining trees from distance matrices.

PROTDIST was also used to calculate distance matrix from different alignments using JTT as the substitution matrix, and gamma model for rate variation.

2.4 MAXIMUM PARSIMONY

PROTPARS program from PHYLIP v.3.67, and PAUP v XXX was used to calculate maximum parsimony trees. ProtPars was used with default settings, and whenever multiple trees were obtained, RF distances were calculated from all the trees and tree with lowest RF distance was selected for average calculations.

For maximum parsimony trees with PAUP* version 4 we used heuristic search with stepwise addition.

2.5 MAXIMUM LIKLIHOOD

Maximum Liklihood trees were generated using PhyML version3.0, RAxML version 7.0.3 and GARLI1.0 (Figure 4) and GARLI 2.0 (Figure 6)

2.5.1RAxML-default

```
raxmlHPC-PTHREADS -s <input file> -n <output suffix> -
m PROTCATWAG -T <no. of Processors>
```

2.5.2 PhyML

Default settings

PhyML v3.0 was used at default settings. Default settings uses, LG as substitution model of choice, a discrete gamma model with 4 rate categories. BioNJ tree was used as initial search tree, with NNI as tree topology search method.

```
PhyML_3.0_macOS_i386 -i <input file> -d aa
```

Default+F

In addition to default settings, empirical values were calculated from data, and used for equilibrium state frequencies.

```
PhyML_3.0_macOS_i386 -i <input file> -d aa -f e
```

2.5.3 GARLI

For results generated in Figure 4, GARLI 1.0 was used with WAG as substitution matrix of choice. Empirical values were used for Equilibrium state frequencies (+F). Gamma Distributed rate categories were used with invariant sites as estimated from data in hand. Two search replicates were used for each tree, and the best tree as generated and calculated by GARLI was used for RF distance calculation.

For results depicted in Figure 6, GARLI 2.0 was used with parameters same as utilized as GARLI1.0, except number of search replicates were increased to 10 instead of 2. Best tree as specified by GARLI was used for RF distance calculation.

Garli-1.0/2.0 -b <configuration file>

```
[general]

datafname = <Input File>

searchreps = 2/10

datatype = aminoacid

ratematrix = WAG

statefrequencies = empirical

ratehetmodel = gamma

numratecats = 4

invariantsites = estimate

[master]

limsprrange = 6

meanbrlenmuts = 5

gammashapebrlen = 1000

gammashapemodel = 1000


bootstrapreps = 0

resampleproportion = 1.0

inferinternalstateprobs = 0

***All other parameters were kept default
```

2.6 Bayesian Analysis

We used parallel version of MrBayes v3.2.12 for our Bayesian analysis. Nexus files with aligned sequences from MUSCLE, were used as inputs and MrBayes was used at 3 different settings:

i) Default settings (2 independent runs) with mixed amino acid substitution model, and gamma model of rate parameters. (200,000-250,000 generations with sampling frequency of 100)

ii) 16 parallel runs with WAG substitution model, and gamma model of rate parameters. (100,000 generations with sampling frequency of 100)

iii) 32 parallel runs with WAG substitution model, and gamma model of rate of rate variation. (200,000 generations with sampling frequency of 100)

Runs were stopped when they reached stationarity as judged by examining log likelihood values of cold chain, and standard deviation of split frequencies. First, 25% samples were discarded as ‘burn-in’, and majority rule consensus trees from the multiple runs were used for RF distance calculation. All three settings tested gave statistically similar results. Trees with best RF distance (among all three settings) for each data set was used to calculate average RF distance, as represented in figure 5.

3. GENERATION OF SIMULATED DATASETS

We used ROSE v1.3 with default settings to generate simulated data sets, at 4 different PAM distances (100, 550, 650, 700). 25 independent replicates were selected at each divergence range. Each dataset was comprised of 100 sequences with an average sequence length of 450 amino acids. Default settings were used for Insertion threshold (0.0005) and Deletion Thresholds (0.0005), substitution matrix (PAM), and the mutation probabilities. Some of the parameters used for generation are show in the configuration file. Sequences and true alignments were recorded in fasta format, and true trees were stored in phylip format. Sequence sets were used as input data sets in PHYRN and other inference methods. True trees were used to calculate the Robinson-Foulds (RF) Distance, while true alignments were used as inputs in experiments shown in figure 6.

We used Seq-Gen v 1.3.2 with PAM as the default substitution matrix and varied scaling factor from 0.1 to 1 to generate multiple replicates (n=25) of the synthetic data sets with sequences at different divergence ranges. The SeqGen scaling factor scales the branch lengths of the input tree to a specified value before generating data set from the input tree. This changes the expected number of amino acid substitutions per site for each branch, and thus changes the overall divergence of the simulated tree. Other settings were kept as defaults during SeqGen simulations.

ROSE Configuration file

```
int SequenceNum = 100;

String TheInsFunc = "[.1,.1,.1,.1,.1,.1,.05,.05,.05,.05,.05,.05,.05,.05]";

String AlignmentFormat = "FASTA";

String OutputFilebase = "test";

String SequenceLen = "450";

String ChooseFromLeaves = "True";

String TreeWithAncestors = "False";

String TheInsertThreshold = "0.00005";

String TheFreq = "[.087,.041,.040,.047,.033,.038,.050,.089,.034,.037,.085,.081,.015,.040,.051,.070,.058,.010,.030,.065]";

String AlignmentSuffix = ".alg";

String SequenceSuffix = ".fa";

String TreeWithSequences = "False";

String TreeSequencesWithGaps = "False";

String SeedVal = "5";

String Relatedness = "<DIVERGENCE RANGE>";

String InputType = "1";

String TheDeleteThreshold = "0.00005";

String ThePAMMatrix = "[[9867.0, 2.0, 9.0, 10.0, 3.0, 8.0, 17.0, 21.0, 2.0, 6.0, 4.0, 2.0, 6.0, 2.0, 22.0, 35.0, 32.0, 0.0, 2.0,
18.0],[ 1.0,9913.0, 1.0, 0.0, 1.0, 10.0, 0.0, 0.0, 10.0, 3.0, 1.0, 19.0, 4.0, 1.0, 4.0, 6.0, 1.0, 8.0, 0.0, 1.0],[ 4.0, 1.0,9822.0, 36.0,
0.0, 4.0, 6.0, 6.0, 21.0, 3.0, 1.0, 13.0, 0.0, 1.0, 2.0, 20.0, 9.0, 1.0, 4.0, 1.0],[ 6.0, 0.0, 42.0,9859.0, 0.0, 6.0, 53.0, 6.0, 4.0, 1.0,
0.0, 3.0, 0.0, 0.0, 1.0, 5.0, 3.0, 0.0, 0.0, 1.0],[ 1.0, 1.0, 0.0, 0.0,9973.0, 0.0, 0.0, 0.0, 1.0, 1.0, 0.0, 0.0, 0.0, 0.0, 1.0, 5.0, 1.0,
0.0, 3.0, 2.0],[ 3.0, 9.0, 4.0, 5.0, 0.0,9876.0, 27.0, 1.0, 23.0, 1.0, 3.0, 6.0, 4.0, 0.0, 6.0, 2.0, 2.0, 0.0, 0.0, 1.0],[ 10.0, 0.0, 7.0,
56.0, 0.0, 35.0,9865.0, 4.0, 2.0, 3.0, 1.0, 4.0, 1.0, 0.0, 3.0, 4.0, 2.0, 0.0, 1.0, 2.0],[ 21.0, 1.0, 12.0, 11.0, 1.0, 3.0, 7.0,9935.0, 1.0,
0.0, 1.0, 2.0, 1.0, 1.0, 3.0, 21.0, 3.0, 0.0, 0.0, 5.0],[ 1.0, 8.0, 18.0, 3.0, 1.0, 20.0, 1.0, 0.0,9912.0, 0.0, 1.0, 1.0, 0.0, 2.0, 3.0, 1.0,
1.0, 1.0, 4.0, 1.0],[ 2.0, 2.0, 3.0, 1.0, 2.0, 1.0, 2.0, 0.0, 0.0,9872.0, 9.0, 2.0, 12.0, 7.0, 0.0, 1.0, 7.0, 0.0, 1.0, 33.0],[ 3.0, 1.0,
3.0, 0.0, 0.0, 6.0, 1.0, 1.0, 4.0, 22.0,9947.0, 2.0, 45.0, 13.0, 3.0, 1.0, 3.0, 4.0, 2.0, 15.0],[ 2.0, 37.0, 25.0, 6.0, 0.0, 12.0, 7.0, 2.0,
2.0, 4.0, 1.0,9926.0, 20.0, 0.0, 3.0, 8.0, 11.0, 0.0, 1.0, 1.0],[ 1.0, 1.0, 0.0, 0.0, 0.0, 2.0, 0.0, 0.0, 0.0, 5.0, 8.0, 4.0,9874.0, 1.0,
0.0, 1.0, 2.0, 0.0, 0.0, 4.0],[ 1.0, 1.0, 1.0, 0.0, 0.0, 0.0, 0.0, 1.0, 2.0, 8.0, 6.0, 0.0, 4.0,9946.0, 0.0, 2.0, 1.0, 3.0, 28.0,
0.0],[ 13.0, 5.0, 2.0, 1.0, 1.0, 8.0, 3.0, 2.0, 5.0, 1.0, 2.0, 2.0, 1.0, 1.0,9926.0, 12.0, 4.0, 0.0, 0.0, 2.0],[ 28.0, 11.0, 34.0, 7.0, 11.0,
4.0, 6.0, 16.0, 2.0, 2.0, 1.0, 7.0, 4.0, 3.0, 17.0,9840.0, 38.0, 5.0, 2.0, 2.0],[ 22.0, 2.0, 13.0, 4.0, 1.0, 3.0, 2.0, 2.0, 1.0, 11.0, 2.0,
8.0, 6.0, 1.0, 5.0, 32.0,9871.0, 0.0, 2.0, 9.0],[ 0.0, 2.0, 0.0, 0.0, 0.0, 0.0, 0.0, 0.0, 0.0, 0.0, 0.0, 0.0, 0.0, 1.0, 0.0, 1.0, 0.0,9976.0,
1.0, 0.0],[ 1.0, 0.0, 3.0, 0.0, 3.0, 0.0, 1.0, 0.0, 4.0, 1.0, 1.0, 0.0, 0.0, 21.0, 0.0, 1.0, 1.0, 2.0,9945.0, 1.0],[ 13.0, 2.0, 1.0, 1.0,
3.0, 2.0, 2.0, 3.0, 3.0, 57.0, 11.0, 1.0, 17.0, 1.0, 3.0, 2.0, 10.0, 0.0, 2.0,9901.0]]";

String TheMutationProbability =
"[1.0,1.0,1.0,1.0,1.0,1.0,1.0,1.0,1.0,1.0,1.0,1.0,1.0,1.0,1.0,1.0,1.0,1.0,1.0,1.0,1.0,1.0,1.0,1.0,1.0,1.0,1.0,1.0,1.0,1.0,1.0,1.0,1.0,1.0,1.0,1.0,1.0,1.0,1.0,1.
0,1.0,1.0,1.0,1.0,1.0,1.0,1.0,1.0,1.0,1.0,1.0,1.0,1.0,1.0,1.0,1.0,1.0,1.0,1.0,1.0,1.0,1.0,1.0,1.0,1.0,1.0,1.0,1.0,1.0,1.0,1.0,1.0,1.0,1.0,1.0,1.0,1.0,1.0,1.0,1.
0,1.0,1.0,1.0,1.0,1.0,1.0,1.0,1.0,1.0,1.0,1.0,1.0,1.0,1.0,1.0,1.0,1.0,1.0,1.0,1.0,1.0,1.0,1.0,1.0,1.0,1.0,1.0,1.0,1.0,1.0,1.0,1.0,1.0,1.0,1.0,1.0,1.0,1.0,1.0,1.
0,1.0,1.0,1.0,1.0,1.0,1.0,1.0,1.0,1.0,1.0,1.0,1.0,1.0,1.0,1.0,1.0,1.0,1.0,1.0,1.0,1.0,1.0,1.0,1.0,1.0,1.0,1.0,1.0,1.0,1.0,1.0,1.0,1.0,1.0,1.0,1.0,1.0,1.0,1.0,1.
0,1.0,1.0,1.0,1.0,1.0,1.0,1.0,1.0,1.0,1.0,1.0,1.0,1.0,1.0,1.0,1.0,1.0,1.0,1.0,1.0,1.0,1.0,1.0,1.0,1.0,1.0,1.0,1.0,1.0,1.0,1.0,1.0,1.0,1.0,1.0,1.0,1.0,1.0,1.0,1.
0,1.0,1.0,1.0,1.0,1.0,1.0,1.0,1.0,1.0,1.0,1.0,1.0,1.0,1.0,1.0,1.0,1.0,1.0,1.0,1.0,1.0,1.0,1.0,1.0,1.0,1.0,1.0,1.0,1.0,1.0,1.0,1.0,1.0,1.0,1.0,1.0,1.0,1.0,1.0,1.
0,1.0,1.0,1.0,1.0,1.0,1.0,1.0,1.0,1.0,1.0,1.0,1.0,1.0,1.0,1.0,1.0,1.0,1.0,1.0,1.0,1.0,1.0,1.0,1.0,1.0,1.0,1.0,1.0,1.0,1.0,1.0,1.0,1.0,1.0,1.0,1.0,1.0,1.0,1.0,1.
0,1.0,1.0,1.0,1.0,1.0,1.0,1.0,1.0,1.0,1.0,1.0,1.0,1.0,1.0,1.0,1.0,1.0,1.0,1.0,1.0,1.0,1.0,1.0,1.0,1.0,1.0,1.0,1.0,1.0,1.0,1.0,1.0,1.0,1.0,1.0,1.0,1.0,1.0,1.0,1.
0,1.0,1.0,1.0,1.0,1.0,1.0,1.0,1.0,1.0,1.0,1.0,1.0,1.0,1.0,1.0,1.0,1.0,1.0,1.0,1.0,1.0,1.0,1.0,1.0,1.0,1.0,1.0,1.0,1.0,1.0,1.0,1.0,1.0,1.0,1.0,1.0,1.0,1.0,1.0,1.
0,1.0,1.0,1.0,1.0,1.0,1.0,1.0,1.0,1.0,1.0,1.0,1.0,1.0,1.0,1.0,1.0,1.0,1.0,1.0,1.0,1.0,1.0,1.0,1.0,1.0,1.0,1.0,1.0,1.0,1.0,1.0,1.0,1.0,1.0,1.0,1.0,1.0,1.0,1.0,1.
0,1.0,1.0,1.0,1.0,1.0,1.0,1.0,1.0,1.0,1.0,1.0,1.0,1.0,1.0,1.0,1.0,1.0,1.0,1.0,1.0,1.0,1.0,1.0,1.0,1.0,1.0,1.0,1.0,1.0,1.0,1.0,1.0,1.0,1.0,1.0,1.0,1.0,1.0,1.0,1.
0,1.0,1.0,1.0,1.0,1.0,1.0,1.0,1.0,1.0,1.0]";

String TheDelFunc = "[.1,.1,.1,.1,.1,.1,.05,.05,.05,.05,.05,.05,.05,.05]";

String TheAlphabet = "ARNDCQEGHILKMFPSTWYV"
```
